# SIMBA: A robust and generalizable measure of data imbalance

**DOI:** 10.1016/j.patter.2025.101395

**Published:** 2025-10-21

**Authors:** Julie R. Pivin-Bachler, Egon L. van den Broek

**Affiliations:** 1 Information and Computing Sciences, Utrecht University, Utrecht 3584 CC, the Netherlands

**Keywords:** data imbalance, data distribution, feature importance, machine learning, measure, benchmark, survey, data complexity, domain generalization, status of imbalance, SIMBA

## Abstract

Ranging from health to cybersecurity, real-world data are heavily imbalanced. Handling imbalance is among the formidable challenges of machine learning (ML), as it deteriorates ML’s performance, yielding biased results toward majority classes. However, finding an adequate measure to assess the impact of data imbalance is a field of research by itself. Following a review of the available imbalance measures, we introduce the status of imbalance (SIMBA), which considers data distribution and overlap, both of which are crucial to assess the impact of imbalance. SIMBA is benchmarked against seven imbalance measures on five ML models, 428 synthetic and 70 non-synthetic datasets from various domains. Resulting correlation coefficients between imbalance measures and classification performance and an analysis with 20 complexity measures prove that SIMBA consistently outperforms other measures. Overall, SIMBA accurately quantifies multiclass data imbalance and may help alleviate ML data imbalance challenges in the future.

## Highlights

• Review of imbalance measures
• Existing imbalance measures do not accurately reflect classification difficulty
• Introduction of SIMBA, a reliable, robust, generic imbalance measure
• SIMBA outperforms existing measures as a classification difficulty indicator

## The bigger picture

Machines can learn via examples provided to them. Real-world data, however, are often imbalanced: certain types of examples appear far more often than rare ones. For instance, in cybersecurity, malicious attacks occur much less frequently than normal network activity, and with medical images, the majority show healthy tissue and only a minority show malignant tissue. This lack of examples for rare types or classes poses a huge challenge to machine learning, as these are the most important cases yet the hardest to learn. In high-stakes applications, where an attack or malignant tissue needs to be correctly classified or recognized, this can mean the difference between life and death.

To date, even machine learning’s newest branches of deep learning (e.g., foundational models) struggle with the lack of data for rare cases. Consequently, rare cases are often simply completely ignored or disregarded, having too little influence on the overall results. As such, data imbalance touches upon the foundations of machine learning and pattern recognition in general. To handle imbalance, several methods have been introduced, but these methods depend on a measure of the extent of imbalance. Until now, a generic data imbalance measure has remained absent and, hence, has limited machine learning’s progress and its application in the real world beyond that of constrained environments.

## Teaser

Data constitute machine learning’s fuel. Unfortunately, real-world data are often imbalanced. This poses a huge challenge to machine learning, as rare cases are hard to learn. So far, no adequate measure has been available to assess the impact of imbalance on machine learning’s performance. As a remedy for this omission, the authors introduce the status of imbalance (SIMBA), a reliable, robust, and generic imbalance measure that outperforms existing ones.

## Introduction

**Figure 1:**
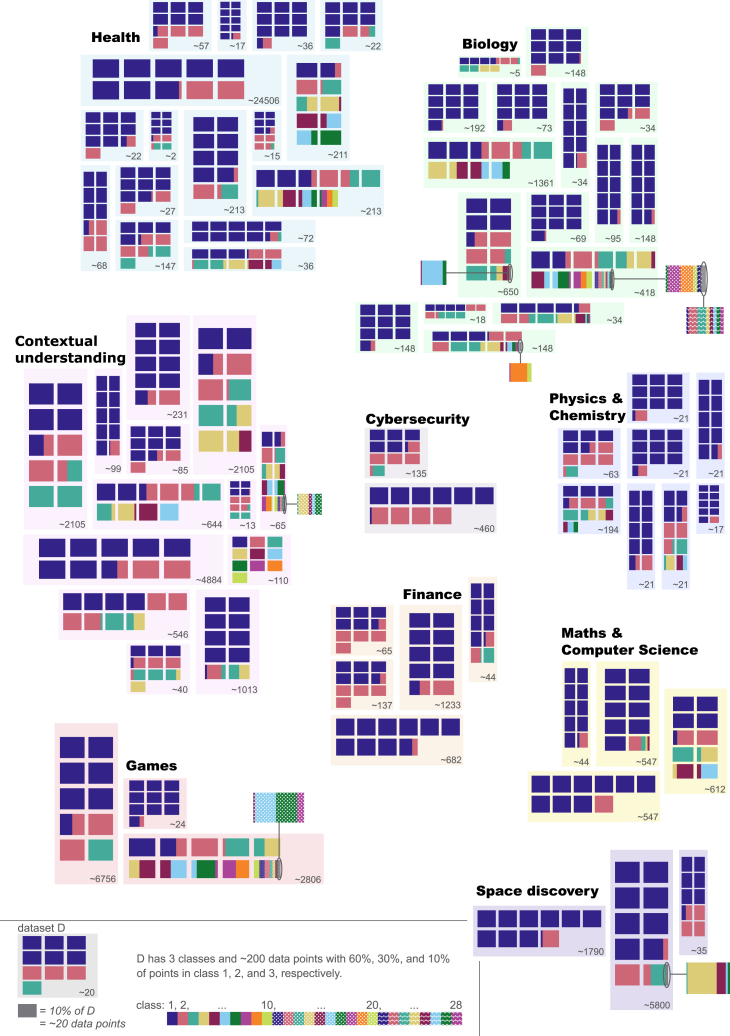
Representation of the 70 non-synthetic datasets used to test the imbalance measures Each dataset is represented as a rectangle with its size and class distribution shown inside it. The datasets are clustered into 9 domains: health, biology, contextual understanding, cybersecurity, physics and chemistry, finance, maths and computer science, games, and space discovery.

Real-world data (e.g., medical data^1^^,^^2^^,^^3^ and cybersecurity^4^^,^^5^^,^^6^) are mostly imbalanced^7^^,^^8^ (see Figure 1), which undermines machine learning (ML)’s performance.^9^^,^^10^ Optimization toward the majority classes is rewarded, with minority classes being neglected.^11^^,^^12^ The scarcity of minority classes’ data has repercussions on ML algorithms’ topologies and their ability to learn reliable patterns for these classes.^13^ This aggravates in the presence of both data overlap, where features of different classes converge on a shared portion of the data space,^14^ and data shifts, such as changes in data characteristics, distributions, and/or labels.^15^ In addition, class imbalance has been shown to introduce biases into predictive models (e.g., ethnicity-based bias in healthcare predictions), resulting in unfair decision-making processes and, thus, unreliable or non-generalizable outcomes.^1^^,^^16^ One of the most notable issues induced by imbalance is a bias toward the majority classes, where these classes get abusively predicted. In many problems, the majority class is the negative class, the one the researchers are not interested in (e.g., network behavior without attack), leading to false negatives.^11^^,^^17^ To “solve” these issues, artificial, forced balanced datasets are often used.^18^^,^^19^ This is either done at the stage of data collection or afterward, via data sampling. Thus, the resulting data do not reflect reality, which renders generalizability uncertain.^15^

To solve the imbalanced data challenge, three main strategies exist^13^^,^^20^^,^^21^: (1) data-level techniques, which adjust distribution via over-/under-sampling (e.g., synthetic minority over-sampling technique [or SMOTE] and its extensions^22^^,^^23^); (2) algorithm-level techniques, which modify learning algorithms to reduce bias; and (3) hybrid techniques that combine both. The choice of strategy depends on the level of imbalance.^24^^,^^25^^,^^26^^,^^27^^,^^28^ Imbalance is defined as “any dataset with an unequal class distribution,”^29^ yet not all imbalance levels hinder the learning process; for example, a 100-95 split in a binary dataset will not be an issue. However, there is no universally accepted definition of what constitutes small, moderate, high, or extreme imbalance, resulting in inconsistencies between papers.^30^

Until now, no measure has existed that fully captures the extent of multiclass data imbalance (see Box 1). Such a measure should effectively reflect the impact of imbalance on ML’s efficiency to determine whether or not any correction is needed and, if so, what method to apply.^12^ Therefore, it should include information on both data distribution and the discriminant power of features.^14^ To address this need, we introduce a robust and generalizable measure: the status of imbalance (SIMBA). SIMBA includes information about (1) data distribution, by comparing the actual distribution to a perfectly balanced one with the statistical log likelihood ratio test, similar to the likelihood ratio imbalance degree (LRID), and (2) data overlap, by incorporating the correlation between features and class labels via the following definition:

(Equation 1) $$\begin{array}{ccc} \text{SIMBA} & = & \frac{-2}{\bar{r}}\sum_{c=1}^{C}\frac{n_{c}}{N}\ln\left(\frac{N}{Cn_{c}}\right)\\ \text{with}\;\bar{r} & = & \frac{1}{C.f^{*}}\sum_{i=1}^{f^{*}}\sum_{c=1}^{C}|PCC\left(f_{i},c\right)|\text{,} \end{array}$$

where, within one dataset, *C* represents the number of classes, *n*_*c*_ the number of points in class *c*, *N* the total number of points, *f*^∗^ the number of non-redundant features, *f*_*i*_ the i^*th*^ non-redundant feature, and *PCC*(*f*_*i*_,*c*) the Pearson correlation coefficient (PCC) between *f*_*i*_ and class *c*.

Box 1 **Summary of existing imbalance measures**

**Figure 2:**
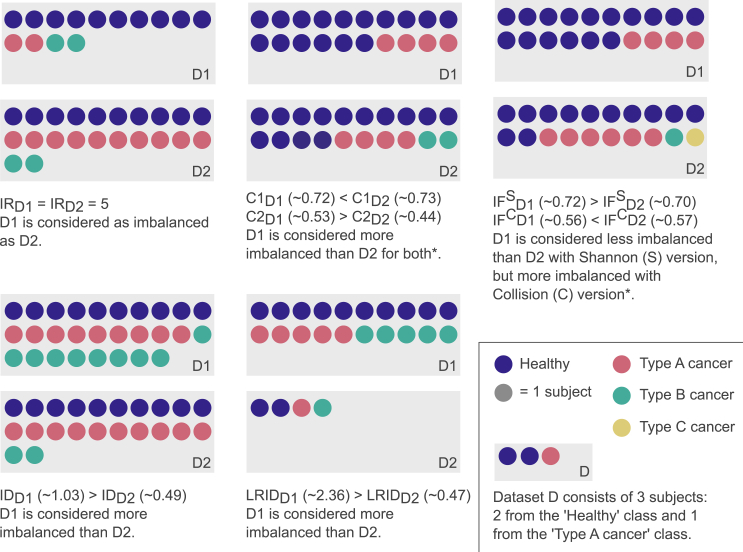
Edge cases of existing imbalance measures Examples for which the imbalance ratio (IR), entropy of class proportions (C1), multiclass imbalance ratio (C2), imbalance degree (ID), likelihood ratio imbalance degree (LRID), and imbalance factor (IF) do not work. Each example has two illustrative synthetic datasets (D1 and D2) with patients who are healthy or who a type A, B, or C cancer. The adjusted IR (Adj-IR) shares the same limitation as IR; thus, it is not represented. ∗For IR, C2, ID, and LRID, a value of 0 means a perfect balance, and increasing values indicate increasing imbalance. In contrast, C1 and IF both range between 0 and 1, with 1 meaning a perfect balance and 0 a total imbalance.

The most widely used measure for imbalance is the imbalance ratio (IR). It corresponds to the ratio of datapoints in the majority class to those in the minority class. The main shortcoming of the IR is that it fails to capture the extent of imbalance in multiclass datasets, which might include several minority classes^25^ (Figure 2). Additionally, the IR focuses solely on class distribution and ignores feature information. However, imbalance impacts classification performance more when facing class overlap, where different classes share similarities in their features.^14^^,^^24^ Even with extreme imbalance, highly discriminant features enable easy classification^31^^,^^32^ (e.g., color distinguishes strawberries from bananas, even in the case of “high” imbalance). Thus, feature information should be considered to have accurate imbalance descriptions, relative to classification difficulty.^14^^,^^29^^,^^32^^,^^33^^,^^34^ The adjusted imbalance ratio (Adj-IR)^35^ improved upon the IR by including information about the number of discriminant features in a dataset. However, it remains unsuitable for multiclass problems.

The lack of imbalance measures applicable to multiclass datasets led to the emergence of 5 measures, namely the entropy of class proportions (C1),^36^^,^^37^ the multiclass imbalance ratio (C2),^37^^,^^38^ the imbalance degree (ID),^39^ the likelihood ratio imbalance degree (LRID),^40^ and the imbalance factor (IF)^41^ (see Table 1). All of these measures have limitations, yielding incoherent results (Figure 2). Notably, the constrained ranges of C1 and C2 can render the comparison between two datasets with a different number of classes incoherent. Similarly, the range of IDs varies depending on the number of minority classes, which gives inconsistent results. LRID is not normalized, leading to some datasets being considered more imbalanced only because they are larger. Finally, in some cases with two datasets with a different number of minority classes, IF results in inconsistencies depending on the version of the measure that is used. Indeed, ID and IF both have one parameter that is left for the researcher to choose, leading to variations in the measure’s formula and its results. Thus, two researchers comparing the same datasets might reach opposite conclusions depending on the version used. As suggested by the papers introducing them, we use the Hellinger distance for ID^39^ and the collision version of IF^41^ throughout this paper unless specified otherwise. For all measures (except C1 and IF), a higher score indicates a more severe imbalance. For C1 and IF, it is the opposite. More details on the imbalance measures’ formulas can be found in the methods.

SIMBA can be applied to datasets with any number of classes, features, and samples. It is domain independent and generalizes to various classifiers and evaluation metrics, making it a robust and generalizable measure. SIMBA’s efficiency is shown through two phases, with (1) synthetic datasets in controlled scenarios and (2) an extensive benchmark, including all measures presented in Box 1 on 70 real datasets covering 9 domains where imbalance is prevalent (Figure 1), 35 binary and 35 multiclass, using 5 common ML classifiers.

In the next section, we explain SIMBA in detail and describe the experiments we conducted to evaluate SIMBA. The subsequent results section presents all experimental outcomes. We end with the discussion, which provides a summary of the results and a reflection on the results and SIMBA in general and identifies its limitations.

## Methods

This section details SIMBA’s formula (Equation 1). Subsequently, the experiments are described to evaluate SIMBA and benchmark it against 7 imbalance measures (Table 1): imbalance ratio (IR), adjusted IR (Adj-IR), entropy of class proportions (C1), multiclass IR (C2), imbalance degree (ID), LRID, and imbalance factor (IF). The benchmark includes synthetic and real datasets, the selection of evaluation metrics and classifiers, and two control studies to evaluate the core components of SIMBA and its link to other data complexity measures.

### The formula for SIMBA

#### Starting from existing imbalance measures

SIMBA is benchmarked against 7 imbalance measures, whose formulas are provided in Table 2. Adj-IR includes *λ*, a parameter controlling the importance of the penalty term for the discriminant power of features. Similar to the paper presenting Adj-IR,^35^ *λ* = 1 is taken for all the experiments. ID values rely on a chosen distance function, *d*(). As suggested by the paper that introduced ID,^39^ the Hellinger distance is selected for *d*(). Finally, IF includes a parameter *α*, the Rényi entropy order. On average, the best results are obtained with *α* = 2 (the limiting case called Collision entropy).^41^ Thus, IF_*Collision*_ is used in all experiments unless specified otherwise. These selected parameters remain constant for all tested datasets to ensure a fair comparison.

**Table 1:** Characteristics of imbalance measures

Measure	Range	Multiclass	Features	#DS
Imbalance ratio (IR)	[0, *∞*[	no	no	N/A
Adjusted imbalance ratio (Adj-IR)	]–*∞*, *∞*[	no	yes	20
Entropy of class proportions (C1)	[0, 1]	yes	no	23
Multiclass imbalance ratio (C2)	[0, 1]	yes	no	31
Imbalance degree (ID)	{0} U [m−1, m]	yes	no	15
Likelihood ratio imbalance degree (LRID)	[0, *∞*[	yes	no	20
Imbalance factor (IF)	[0, 1]	yes	no	15
Status of imbalance (SIMBA)	[0, *∞*[	yes	yes	70

Comparison of range, ability to handle multiclass problems, taking into account features, and the number of datasets (#DS) the imbalance measures were tested on.

**Table 2:** Formulas of the 7 existing imbalance measures

Measure	Formula	Additional information
Imbalance ratio (IR)	$\text{IR}=\frac{\max\left(\left\{n_{1},n_{2},\ldots ,n_{C}\right\}\right)}{\min\left(\left\{n_{1},n_{2},\ldots ,n_{C}\right\}\right)}$	–
Adjusted imbalance ratio (Adj-IR)	$\text{Adj-IR}=\text{IR}-\lambda \;\log\left(f_{d}\right)$	*λ* is a constant parameter controlling the importance of the penalty term, and *f*_*d*_ is the number of discriminant features, i.e., the number of features having a non-zero correlation with the labels with *p* < 0.05
Entropy of class proportions (C1)	$C1=-\frac{1}{\log\left(C\right)}\sum_{c=1}^{C}{\hat{p}}_{c}\;\log\left({\hat{p}}_{c}\right)$	corresponds to IF for *α* = 1, also known as IF_*Shannon*_
Multiclass imbalance ratio (C2)	$C2=1-\frac{1}{IR_{m}}$,	–
	with $IR_{m}=\frac{C-1}{C}\sum_{c=1}^{C}\frac{n_{c}}{N-n_{c}}$	
Imbalance degree (ID)	$\text{ID}=\frac{d\left(\hat{p},b\right)}{d\left(p_{m},b\right)}+\left(m-1\right)$	*d*() is a distance function to be chosen, *m* the number of minority classes, and *p*_*m*_ the distribution of *D* with *m* minority classes with the highest distance to *b* (i.e., worst case of imbalance with *m* minority classes); *p*_*m*_ is attained when the *m* minority classes contain no datapoints
Likelihood ratio imbalance degree (LRID)	$\text{LRID}=-2\sum_{c=1}^{C}n_{c}\;\ln\left(\frac{b_{c}}{{\hat{p}}_{c}}\right)$	–
	$\text{LRID}=-2\sum_{c=1}^{C}n_{c}\;\ln\left(\frac{N}{Cn_{c}}\right)$	–
Imbalance factor (IF)	$\text{IF}=\frac{\frac{1}{1-\alpha }\log\left(\sum_{c=1}^{C}{\hat{p}}_{c}^{\alpha }\right)}{\log\left(C\right)}$	*α* is the Rényi entropy order, left to the researcher to choose
	${\text{IF}}_{Collision}=\frac{-\log\left(\sum_{c=1}^{C}{\hat{p}}_{c}^{2}\right)}{\log\left(C\right)}$	for *α* = 2

For a dataset *D*, *C* is the total number of classes, *n*_*c*_ the number of datapoints in class *c*, and *N* the total number of points in *D*. If *D* is perfectly balanced, $b_{c}=\frac{1}{C}$ is the proportion of each class *c* and *b* = {*b*_1_, *b*_2_, …, *b*_*C*_} is the distribution for all *C* classes. In contrast, ${\hat{p}}_{c}=\frac{n_{c}}{N}$ is the observed distribution—hence, the estimated data distribution—for each class in the dataset (for a balanced dataset, $b_{c}={\hat{p}}_{c}$), and $\hat{p}=\left\{{\hat{p}}_{1},{\hat{p}}_{2},\ldots ,{\hat{p}}_{C}\right\}$ is the observed distribution for all *C* classes.

The choice of *α* for IF leads to different results, to the point of changing the conclusion when comparing the imbalance of two datasets (Figure 2). To prevent SIMBA from leading to conflicting conclusions, its formula originates from LRID, an imbalance measure applicable to multiclass problems. Following LRID, SIMBA does not require researcher-selected parameters and facilitates an easy fix with borderline examples, as presented in Figure 2. Indeed, the main critique of LRID is the lack of normalization and the lack of feature information, both of which are included in SIMBA’s formula.

LRID is based on the statistical log likelihood ratio test, which examines the differences between two distributions.^42^ In the context of imbalance, LRID assesses how much the observed data distribution differs from the ideal balanced distribution^35^ via the formula

(Equation 2) $$\text{LRID}=-2\sum_{c=1}^{C}n_{c}\;\ln\left(\frac{b_{c}}{{\hat{p}}_{c}}\right)=-2\sum_{c=1}^{C}n_{c}\;\ln\left(\frac{N}{Cn_{c}}\right)\text{,}$$

with *C* representing the total number of classes, *n*_*c*_ the number of datapoints in class *c*, $b_{c}=\frac{1}{C}$ the ideal proportion of class *c* to obtain a balanced dataset, ${\hat{p}}_{c}=\frac{n_{c}}{N}$ the observed distribution (i.e., the estimated data distribution) for class *c*, and *N* the total number of points in the dataset.

In the case of a balanced dataset, $b_{c}={\hat{p}}_{c}$ gives LRID = 0. Otherwise, LRID’s value increases as the difference between *b*_*c*_ and ${\hat{p}}_{c}$ gets larger. Thus, LRID ranges between 0 and +*∞*, with larger values indicating a greater imbalance extent.

#### Normalization

Two datasets with an equal proportion (in %) of points in each class should yield the same result with an imbalance measure solely taking data distribution into account. Let us take 2 datasets, *DS*_1_ and *DS*_2_, with 3 classes, each with the following distribution of points: *DS*_1_: (*n*_1_, *n*_2_, *n*_3_), with *n*_*i*_ as the number of points in class {*i*}, and *DS*_2_: (*an*_1_, *an*_2_, *an*_3_), with $a,n_{i}\in N^{*}$. Let us assume that *DS*_1_ and *DS*_2_ are not perfectly balanced; therefore, ${\text{LRID}}_{DS_{1}}\neq 0$, and ${\text{LRID}}_{DS_{2}}\neq 0$. Let us write *N*_1_ = *n*_1_ + *n*_2_ + *n*_3_ and *N*_2_ = *an*_1_ + *an*_2_ + *an*_3_ = *aN*_1_, the total number of points in *DS*_1_ and *DS*_2_, respectively. Then, by applying Equation 2, we obtain

$${\text{LRID}}_{DS_{1}}=2\sum_{c=1}^{3}n_{c}\;\ln\left(\frac{N_{1}}{3n_{c}}\right)=-2\left[n_{1}\;\ln\left(\frac{N_{1}}{3n_{1}}\right)+n_{2}\;\ln\left(\frac{N_{1}}{3n_{2}}\right)+n_{3}\;\ln\left(\frac{N_{1}}{3n_{3}}\right)\right]{\text{LRID}}_{DS_{2}}=-2\left[an_{1}\;\ln\left(\frac{aN_{1}}{3an_{1}}\right)+an_{2}\;\ln\left(\frac{aN_{1}}{3an_{2}}\right)+an_{3}\;\ln\left(\frac{aN_{1}}{3an_{3}}\right)\right]=-2a\left[n_{1}\;\ln\left(\frac{N_{1}}{3n_{1}}\right)+n_{2}\;\ln\left(\frac{N_{1}}{3n_{2}}\right)+n_{3}\;\ln\left(\frac{N_{1}}{3n_{3}}\right)\right]=a\cdot {\text{LRID}}_{DS_{1}}\text{.}$$

As $a\in N^{*}$ and LRID > 0, ${\text{LRID}}_{DS_{2}}>{\text{LRID}}_{DS_{1}}$. *DS*_2_ is considered more imbalanced than *DS*_2_. As our datasets have the same proportion of points in each class, this result is incoherent. Normalizing LRID, which we denote as *N*_LRID, gives

(Equation 3) $$N\_\text{LRID}=-2\sum_{c=1}^{C}\frac{n_{c}}{N}\ln\left(\frac{N}{Cn_{c}}\right)\text{.}$$

Subsequently, with Equation 3, we obtain

$$\begin{array}{ccc} N\_{\text{LRID}}_{DS_{1}} & = & \frac{-2}{N_{1}}\left[n_{1}\;\ln\left(\frac{N_{1}}{3n_{1}}\right)+n_{2}\;\ln\left(\frac{N_{1}}{3n_{2}}\right)+n_{3}\;\ln\left(\frac{N_{1}}{3n_{3}}\right)\right]\\ N\_{\text{LRID}}_{DS_{2}} & = & \frac{-2}{aN_{1}}\left[an_{1}\;\ln\left(\frac{aN_{1}}{3an_{1}}\right)+an_{2}\;\ln\left(\frac{aN_{1}}{3an_{2}}\right)+an_{3}\;\ln\left(\frac{aN_{1}}{3an_{3}}\right)\right]\\ & = & \frac{-2}{N_{1}}\left[n_{1}\;\ln\left(\frac{N_{1}}{3n_{1}}\right)+n_{2}\;\ln\left(\frac{N_{1}}{3n_{2}}\right)+n_{3}\;\ln\left(\frac{N_{1}}{3n_{3}}\right)\right]\\ & = & N\_{\text{LRID}}_{DS_{1}} \end{array}$$

With *N*_LRID, the same proportion of points in each class for two datasets of different sizes results in the same imbalance extent, which shows the need for normalization.

#### The discriminant power of features

An imbalance measure should account for feature information to reflect classification difficulty.^14^^,^^32^ With that line of thought, Zhu et al.^35^ created Adj-IR, which adjusts the IR by considering how many features have a non-zero correlation with class labels. However, Adj-IR only applies to binary problems and does not consider feature redundancy. In contrast, SIMBA includes both feature importance and feature redundancy.

#### Feature importance

The correlation between feature values and class labels is a good indicator of feature importance.^35^^,^^43^ In a multiclass problem, a feature can help distinguish one class but not necessarily all of them. We define the average correlation $\bar{r}$ between each class and each non-redundant feature (see next subsection for the definition of a non-redundant feature):

(Equation 4) $$\begin{array}{l} \bar{r}=\frac{1}{C\cdot f^{*}}\sum_{i=1}^{f^{*}}\sum_{c=1}^{C}|PCC\left(f_{i},c\right)|\text{,} \end{array}$$

with *C* representing the number of classes, *f*^∗^ the number of non-redundant features, *f*_*i*_ the i^*th*^ non-redundant feature, and *PCC*(*f*_*i*_,*c*) the PCC between *f*_*i*_ and class *c*.

With LRID, a value of 0 represents perfect balance, while higher values indicate increasing imbalance. To preserve coherence, SIMBA’s value should increase as $\bar{r}$ diminishes, as the less informative the features are, the greater the impact of imbalance gets, and vice versa. Thus, $\bar{r}$ is included in SIMBA’s formula as a dividing factor, which gives

(Equation 5) $$\begin{array}{ccc} \text{SIMBA} & = & \frac{1}{\bar{r}}N\_\text{LRID}\\ & = & \frac{-2}{\bar{r}}\sum_{c=1}^{C}\frac{n_{c}}{N}\ln\left(\frac{N}{Cn_{c}}\right)\\ \;\text{with}\;\bar{r} & = & \frac{1}{C.f^{*}}\sum_{i=1}^{f^{*}}\sum_{c=1}^{C}|PCC\left(f_{i},c\right)|\text{.} \end{array}$$

When $\bar{r}$ tends to 0, SIMBA tends to +*∞*. Theoretically, $\bar{r}=0$ means that none of the features gives any information on any of the classes. Such a dataset would result in an impossible classification task; thus, having SIMBA = +*∞* is coherent. However, in practice, it is very unlikely that $\bar{r}=0$ will be obtained for a given dataset.

To make sure SIMBA correctly takes into account feature information, let us take 2 datasets, *DS*_1_ and *DS*_2_, each with the same number of classes *C* and only 1 feature *f*_1_. Both datasets have the same data distribution (*n*_1_, …, *n*_*C*_). The datasets are not perfectly balanced, thus $\exists \left(i,j\right)\in N^{*},\text{such}\;\text{that}\;n_{i}\neq n_{j}$. Each dataset has a unique feature, so *f*^∗^ = 1, and Equation 4 becomes

$$\begin{array}{l} \bar{r}=\frac{1}{C}\sum_{c=1}^{C}|PCC\left(f_{1},c\right)|\text{.} \end{array}$$

We pose ${\bar{r}}_{DS_{1}}=a$ and ${\bar{r}}_{DS_{2}}=b$, with (*a*,*b*)∈]0, 1]. Let us assume that *f*_1_ holds less discriminant information in *DS*_1_ than in *DS*_2_. Mathematically, this means that ${\bar{r}}_{DS_{1}}<{\bar{r}}_{DS_{2}}$; thus, *a* < *b*. We can write ∃*α* > 1, such that *b* = *α* · *a*. For each dataset, Equation 5 gives

$$\begin{array}{c} {\text{SIMBA}}_{DS_{1}}=\frac{-2}{a}\sum_{c=1}^{C}\frac{n_{c}}{N}\ln\left(\frac{N}{Cn_{c}}\right)\;\text{and}\\ {\text{SIMBA}}_{DS_{2}}=\frac{-2}{\alpha \;\cdot \;a}\sum_{c=1}^{C}\frac{n_{c}}{N}\ln\left(\frac{N}{Cn_{c}}\right)\text{.} \end{array}$$

${\text{SIMBA}}_{DS_{1}}=\alpha \cdot {\text{SIMBA}}_{DS_{2}}$, with *α* > 1; hence, ${\text{SIMBA}}_{DS_{1}}>{\text{SIMBA}}_{DS_{2}}$. Therefore, if a dataset has features holding less discriminant information $\left({\bar{r}}_{DS_{1}}<{\bar{r}}_{DS_{2}}\right)$, then the imbalance measure gets larger than that of another dataset with the same data distribution but more informative features.

#### Redundancy

Desirable features have a strong correlation with the targeted classes but not with each other.^43^^,^^44^^,^^45^ Hence, when calculating the $\bar{r}$ term, only non-redundant features are considered. Two redundant features are usually defined as “highly correlated features.” Indeed, only highly correlated features are considered damaging for classification tasks, not moderately correlated ones.^43^ A correlation coefficient whose absolute value ranges between 0.9 and 1.0 indicates a very strong correlation.^46^ Thus, we consider that two features are redundant if their PCC is larger than 0.9. If this happens, the feature from the pair with the lowest feature importance is removed, while the other one is kept as one of the *f*^∗^ non-redundant features. All correlation and feature importance calculations are performed solely on the training data.

The aim of the $\bar{r}$ term in SIMBA’s formula (Equation 5) is to represent the average information held by each feature to distinguish between classes. If, instead of taking the number of non-redundant features *f*^∗^, the total number of features *f* was taken, $\bar{r}$ would not accurately represent the average information held by the feature set. Let us take dataset *DS*_1_ with *C* classes and 3 features, *f*_1_, *f*_2_, and *f*_3_, such that

$$\frac{1}{C}\sum_{c=1}^{C}|PCC\left(f_{1},c\right)|=0.9,\frac{1}{C}\sum_{c=1}^{C}|PCC\left(f_{2},c\right)\left|=0.9,\text{and}\;\frac{1}{C}\sum_{c=1}^{C}|PCC\left(f_{3},c\right)\right|=0.1\text{.}$$

Now, let us assume that *f*_1_ = *f*_2_, the most extreme case of redundancy. If we consider all features, Equation 5 gives

$$\bar{r}=\frac{1}{3}\sum_{i=1}^{3}\frac{1}{C}\;\sum_{c=1}^{C}|PCC\left(f_{i},c\right)|=\frac{1}{3}\left[0.9+0.9+0.1\right]=0.63\text{,}\text{and}\;\text{so}\;\text{SIMBA}\;\approx \;3.17\sum_{c=1}^{C}\frac{n_{c}}{N}\ln\left(\frac{N}{Cn_{c}}\right)\text{.}$$

However, if we consider only non-redundant features, Equation 5 gives

$$\bar{r}=\frac{1}{2}\sum_{i=1}^{2}\frac{1}{C}\;\sum_{c=1}^{C}|PCC\left(f_{i},c\right)|=\frac{1}{2}\left[0.9+0.1\right]=0.5\text{,}\text{and}\;\text{so}\;\text{SIMBA}=4\sum_{c=1}^{C}\frac{n_{c}}{N}\ln\left(\frac{N}{Cn_{c}}\right)\text{.}$$

In the first case, SIMBA is smaller, indicating a better balance, only because the redundant feature is counted twice in the average degree of discriminant power of features. As redundant features hold very similar information and do not lead to better discrimination between classes, it is better not to count them multiple times and only consider non-redundant features for $\bar{r}$.

### Experiments

The 8 imbalance measures, IR, Adj-IR, C1, C2, ID, LRID, IF, and SIMBA, are tested on both synthetic and real data. Synthetic datasets, generated with the make_classification function from scikit-learn, are used to compare the behavior of each measure in controlled scenarios. Both synthetic and real datasets are used to compare the accuracy of the imbalance measures.

#### Synthetic data

Synthetic datasets are necessary to create scenarios in which data distribution and data overlap are controlled, allowing for the analysis of how each imbalance measure responds to these variations. Artificially generated datasets allow for the examination of results produced by the imbalance measures (1) when the data distribution changes while features remain fixed and (2) when the data distribution is fixed while feature components vary. Thus, to compare the imbalance measures’ behavior on these two aspects separately, 3 scenarios with variations in data distribution and 3 with variations in feature components are used. All measures are compared for data distribution, while only Adj-IR and SIMBA are compared for feature distribution, as they consider feature information.

#### Variations in data distribution

For data distribution, similar scenarios to those introduced by Pirizadeh et al.^41^ are used. The 3 scenarios involve a dataset with 3 classes with a distribution (*n*_1_, *n*_2_, *n*_3_), where *n*_*j*_ represents the number of points in the *j*^*th*^ class. For all 3 scenarios, *i* varies from 5 to 100, resulting in 95 datasets per scenario. The number of features is fixed to 10, among which 2 are informative and non-redundant (i.e., 2 discriminant features that are not highly correlated to each other) and 8 are not informative (i.e., non-discriminant features). By default, the make_classification function from scikit-learn uses a total of 20 features, among which 2 are informative. A total number of 20 features avoids generating “toy” datasets; that is, datasets that would result in a good classification performance regardless of the data distribution among the different classes. However, to prevent issues about dimensionality, the total number of features was reduced to 10, which remains sufficient to prevent the generation of toy datasets.

The scenarios are as follows.

(1) Scenario 1: (100, 5, *i*).
(2) Scenario 2: (50, 50, *i*).
(3) Scenario 3: (10*i*, 50*i*, 100*i*).

Each scenario triggers a different trend. In scenario 1, as *i* increases, the imbalance decreases, although a perfect balance is never reached because of the 2^nd^ class. In scenario 2, the imbalance decreases until *i* = 50, where perfect balance is reached; then, it decreases again when *i* continues increasing. Finally, in scenario 3, regardless of the value of *i*, the proportion of points in each class relative to the dataset’s size remains the same. Thus, the level of imbalance remains the same. As 5-fold cross-validation is used, we take at least 5 datapoints in the minority class for varying data distribution.

#### Variations in feature components

For feature components, three categories are considered. (1) Informative features: discriminant features useful for distinguishing between classes. (2) Redundant features: features that hold the same information for the classes, with 2 features being considered redundant if their absolute correlation is >0.9. (3) Non-informative features: features that do not contribute to class differentiation.

The 3 scenarios involve a dataset with 3 classes with a fixed distribution of (400, 75, 25). The numbers of informative features, redundant features, and non-informative features, noted as (info, rdd, $\bar{\text{info}}$), are the focus of the following scenarios.

(1) Scenario 1: (*i*, 0, 50-*i*), with *i* ranging from 2 to 50, resulting in 48 datasets.
(2) Scenario 2: (5, *i*, 0), with *i* ranging from 0 to 45, resulting in 46 datasets.
(3) Scenario 3: (50-*i*, *i*, 0), with *i* ranging from 0 to 48, resulting in 49 datasets.

We are limited by the make_classification function from scikit-learn, which enforces a minimum of 2 informative features in all scenarios.

In scenario 1, the number of informative features out of 50 features increases with *i*. Intuitively, the more informative features there are, the easier the classification becomes. Thus, as *i* increases, the imbalance impact decreases. In contrast, in scenario 2, 5 features are informative, and as *i* increases, redundant features are added. These features do not bring additional information, so the extent of the imbalance should stay constant, regardless of *i*. Scenario 3 presents a ratio between the number of informative features and the number of redundant features. All features hold information (0 non-informative features), but some are redundant. As *i* increases, the number of informative features decreases, while the number of redundant features increases. In this case, it is hard to predict if the classification task will get easier or more difficult as *i* increases. On the one hand, the more informative features there are, the better. On the other hand, the curse of dimensionality tells us that more features can lead to a more challenging classification process,^47^ which could lead the imbalance to have a stronger effect with a larger number of informative features.

#### Real data

Typically, new imbalance measures are introduced with tests on 15–20 real datasets (Table 1). For the correlation test, the null hypothesis is that the correlation between imbalance measures and evaluation metrics is negligible (absolute value up to 0.1), while the alternative hypothesis is that a strong correlation exists between both (absolute value larger than 0.7).^46^ We take a significance level *α* = 0.01 and aim for a power *β* ≥ 0.9 for both PCC and Spearman’s rank correlation coefficient (SRCC). A power analysis finds that 35 is the minimum number of datasets required for *β* = 0.9. To investigate PCC and SRCC for binary and multiclass problems, both together and separately, we need both 35 binary and 35 multiclass datasets, resulting in a total of 70 datasets.

Similar to other imbalance measure research, the KEEL Dataset Repository^48^ and the UCI Machine Learning Repository^49^ are used to find datasets. The 70 datasets are shown in Figure 1 and described in Table 3. The datasets have 2–28 classes, 3–520 features, and 24–245,057 samples. More details can be found in Table S1. For each dataset, all categorical variables were encoded into integers, and all rows with missing values for one feature or more were removed (this is accounted for in the number of samples reported in Table 3).

**Table 3:** Description of the 70 datasets used in the study

Dataset	#c	#ft	Size	IR (Adj-IR)	C1	C2	ID	LRID	IF	SIMBA
abalone^a^	28	8	4,177	689.0 (686.0)	0.75	0.07	19.65	6,979.63	0.68	18.07
abalone_20_^b^	2	8	1,916	72.69 (69.88)	0.1	0.97	0.86	2,380.9	0.04	11.68
abalone_9–18_^b^	2	8	731	50.0 ± 44.25	0.32	0.88	0.7	691.88	0.17	4.69
adult^a^	2	14	48,842	3.18 (−0.41)	0.79	0.43	0.36	13,958.71	0.65	2.2
balance^a^	3	4	625	5.88 (3.88)	0.83	0.16	0.53	231.2	0.77	1.38
banknote^a^	2	4	1,372	1.25 (−0.34)	0.99	0.02	0.07	16.87	0.98	0.04
bankruptcy^a^	2	95	6,819	30.0 (24.11)	0.21	0.93	0.78	7,509.43	0.09	16.64
breastcancer^a^	2	30	569	1.68 (−2.96)	0.95	0.12	0.17	37.36	0.91	0.18
cardio_10_^a^	10	21	2,126	10.92 (6.53)	0.88	0.08	5.41	1,204.17	0.79	3.83
cardio_3_^a^	3	21	2,126	9.4 (5.16)	0.61	0.6	1.51	1,800.06	0.42	4.63
chess^a^	18	6	28,056	168.63 (166.04)	0.84	0.05	9.57	25,894.22	0.78	15.22
cleveland_0_*vs*_4_^b^	2	13	173	12.31 (8.85)	0.38	0.84	0.66	147.53	0.22	3.52
connect-4^a^	3	42	67,557	6.9 (1.85)	0.77	0.36	1.4	34,325.85	0.63	20.26
contraceptive^a^	3	9	1,473	1.89 (−0.92)	0.97	0.04	0.2	93.79	0.95	0.6
credit^a^	2	15	653	1.21 (−2.38)	0.99	0.02	0.06	5.71	0.99	0.04
dermatology^a^	6	34	358	5.55 (1.09)	0.94	0.05	2.29	77.68	0.89	0.89
dermatology_6_^b^	2	34	358	16.9 (12.44)	0.31	0.88	0.71	342.04	0.16	5.33
drybean^a^	7	16	13,611	6.79 (2.79)	0.94	0.04	3.28	3,033.31	0.9	0.93
ecoli^a^	8	7	336	71.5 (68.92)	0.73	0.2	4.61	377.88	0.63	5.49
ecoli_0_^b^	2	7	336	10.59 (8.0)	0.42	0.81	0.63	268.28	0.25	3.36
ecoli_1_^b^	2	7	336	3.36 (1.78)	0.78	0.45	0.37	104.08	0.63	1.38
glass^a^	6	9	214	8.44 (5.64)	0.84	0.14	3.4	121.17	0.74	3.43
glass_0,1,5_*vs*_2_^b^	2	9	172	9.12 (9.12)	0.47	0.78	0.6	127.5	0.28	10.98
glass_2_^b^	2	9	214	11.59 (10.59)	0.4	0.83	0.65	177.94	0.23	11.74
glass_4_^b^	2	9	214	15.46 (13.14)	0.33	0.87	0.69	198.65	0.17	5.26
glass_5_^b^	2	9	214	22.78 (20.78)	0.25	0.91	0.75	222.01	0.12	8.11
glass_6_^b^	2	9	214	6.38 (3.79)	0.57	0.69	0.53	126.86	0.39	1.83
hayes-roth^a^	3	4	132	1.7 (0.12)	0.98	0.03	0.19	7.14	0.96	0.27
htru2^a^^,^^50^	2	8	17,898	9.92 (6.92)	0.44	0.8	0.62	13,852.4	0.26	1.49
ionosphere^a^	2	34	351	1.79 (−2.86)	0.94	0.15	0.19	28.31	0.89	0.45
knowledge^a^	4	5	403	2.58 (0.26)	0.96	0.04	0.31	42.92	0.94	0.52
landsat^a^	6	36	6,435	2.45 (−2.72)	0.96	0.03	2.23	906.95	0.93	0.43
led7digit^b^	2	7	443	10.97 (8.65)	0.41	0.82	0.64	359.59	0.24	3.11
lenses^a^	3	3	24	3.75 (3.75)	0.84	0.3	1.32	8.61	0.7	1.01
loc_build^a^^,^^51^	3	520	21,048	1.77 (−7.01)	0.97	0.06	1.15	1,538.04	0.93	0.56
loc_floor^a^^,^^51^	5	520	21,048	4.79 (−3.74)	0.94	0.04	0.48	3,804.36	0.92	3.81
lymphography^a^	4	18	148	40.5 (37.04)	0.61	0.32	1.73	158.46	0.54	5.67
new-thyroid^b^	3	5	215	5.0 (4.0)	0.75	0.44	1.4	119.17	0.57	1.35
new-thyroid_1_^b^	2	5	215	5.14 (2.82)	0.64	0.63	0.48	107.02	0.46	1.09
obesity^a^	7	16	2,111	1.29 (−2.52)	1.0	0.0	4.04	14.0	1.0	0.05
page-blocks_0_^b^	2	10	5,472	8.79 (5.47)	0.48	0.78	0.6	3,976.52	0.29	4.12
pageblocks^b^	5	10	5,473	175.46 (172.29)	0.27	0.86	3.75	12,795.53	0.13	15.06
penbased^b^	10	16	1,100	1.1 (−2.71)	1.0	0.0	4.02	1.78	1.0	0.01
${\text{poker}}_{9\_vs\_7}$^b^	2	10	244	29.5 (27.92)	0.21	0.93	0.78	267.84	0.09	10.82
purchase^a^	2	17	12,330	5.46 (1.65)	0.62	0.65	0.49	6,468.21	0.44	4.77
room^a^^,^^52^	4	16	10,129	17.93 (13.93)	0.49	0.71	2.59	14,203.62	0.29	3.62
segment_0_^b^	2	19	2,308	6.02 (1.93)	0.59	0.68	0.52	1,309.03	0.4	4.36
shuttle^a^	7	7	58,000	4,558.6 (4,556.28)	0.34	0.7	4.87	148,512.48	0.23	21.88
skin^a^	2	3	245,057	3.82 (2.23)	0.74	0.51	0.41	89,430.53	0.58	1.4
soybean^a^	4	35	47	1.7 (−1.62)	0.98	0.03	2.12	2.88	0.95	0.31
spambase^a^	2	57	4,601	1.54 (−4.27)	0.97	0.09	0.14	208.19	0.94	0.28
spect-heart^a^	2	22	267	3.85 (−0.6)	0.73	0.51	0.41	98.55	0.57	1.68
steel^a^	7	27	1,941	12.24 (7.84)	0.86	0.11	3.43	1,067.34	0.77	3.42
student^a^	17	30	649	104.0 (99.61)	0.85	0.05	10.5	536.87	0.8	16.84
theorem^a^	6	51	6,118	5.26 (−0.07)	0.89	0.13	3.32	2,416.81	0.78	7.58
thyroid^b^	3	21	720	39.18 (36.85)	0.28	0.88	1.73	1,131.13	0.14	24.65
vehicle_0_^b^	2	18	846	3.25 (−0.45)	0.79	0.44	0.36	249.8	0.64	1.82
vowel_0_^b^	2	13	988	9.98 (7.17)	0.44	0.8	0.62	766.86	0.26	5.1
wallfollowing^a^	4	24	5,456	6.72 (2.55)	0.86	0.14	1.42	2,158.41	0.78	3.19
webphishing^a^	3	9	1,353	6.82 (3.65)	0.82	0.19	0.54	530.52	0.75	1.63
wholesale^a^	3	7	440	6.72 (6.72)	0.71	0.48	1.44	278.91	0.53	14.68
wine^a^	3	13	178	1.48 (−2.11)	0.99	0.02	1.09	4.48	0.98	0.06
wine-quality^b^	7	11	6,497	567.2 (563.88)	0.65	0.24	3.73	8,734.31	0.57	23.53
winequality_*red*_^b^	2	11	691	68.1 (67.1)	0.11	0.97	0.85	853.36	0.04	20.44
wisconsin^b^	2	9	683	1.86 (−1.31)	0.93	0.17	0.2	62.49	0.88	0.13
yeast^a^	10	8	1,484	92.6 (89.6)	0.75	0.15	5.6	1,710.63	0.65	10.33
${\text{yeast}}_{1,2,8,9\_vs\_7}$^b^	2	8	947	30.57 (28.57)	0.2	0.93	0.78	1,046.66	0.09	14.21
yeast_3_^b^	2	8	1,484	8.1 (6.1)	0.5	0.76	0.58	1,029.81	0.31	5.5
yeast_4_^b^	2	8	1,484	28.1 (26.1)	0.22	0.93	0.77	1,613.23	0.1	10.83
yeast_5_^b^	2	8	1,484	32.73 (30.73)	0.19	0.94	0.79	1,660.97	0.09	7.92

The number of classes (#c), features (#ft), and samples (size) and the results with 8 imbalance measures—imbalance ratio (IR), adjusted imbalance ratio (Adj-IR), entropy of class proportions (C1), multiclass imbalance ratio (C2), imbalance degree (ID), likelihood ratio imbalance degree (LRID), imbalance factor (IF), and status of imbalance (SIMBA)—are included. See Table S1 for additional information.

a UCI Machine Learning Repository.^49^

b KEEL Dataset Repository.^48^

#### Control analyses

SIMBA’s formula (Equation 5) contains three core components: (1) normalization, (2) feature importance, and (3) feature redundancy. To evaluate the importance of each component individually, an ablation study is performed on the real data where each component is removed from the formula separately. This results in 4 versions of SIMBA: SIMBA as described in Equation 5; SIMBA without normalization:

$$\begin{array}{c} {\text{SIMBA}}_{NoNorm}=\frac{-2}{\bar{r}}\sum_{c=1}^{C}n_{c}\;\ln\left(\frac{N}{Cn_{c}}\right)\text{,}\\ \text{with}\;\bar{r}=\frac{1}{C.f^{*}}\sum_{i=1}^{f^{*}}\sum_{c=1}^{C}|PCC\left(f_{i},c\right)|\text{;} \end{array}$$

SIMBA without feature importance:

$${\text{SIMBA}}_{NoFtImp}=-2\sum_{c=1}^{C}\frac{n_{c}}{N}\ln\left(\frac{N}{Cn_{c}}\right)\text{;}$$

and SIMBA without feature redundancy:

$$\begin{array}{c} {\text{SIMBA}}_{NoFtRed}=\frac{-2}{\bar{r}}\sum_{c=1}^{C}\frac{n_{c}}{N}\ln\left(\frac{N}{Cn_{c}}\right)\text{,}\\ \;\text{with}\;\bar{r}=\frac{1}{C.f}\sum_{i=1}^{f}\sum_{c=1}^{C}|PCC\left(f_{i},c\right)|\text{,} \end{array}$$

where *f*, the total number of features, replaces *f*^∗^, the total number of non-redundant features. The results of the 4 different versions of SIMBA allow us to analyze the effect of each component separately.

SIMBA focuses on the impact of imbalance on classification performance. As such, it constitutes one aspect of data complexity, but it does not encompass all its dimensions. According to Lorena et al.,^37^ data complexity measures can be grouped into 6 categories: feature based, linearity, neighborhood, network, dimensionality, and class imbalance. SIMBA is a class-imbalance measure. To ensure that SIMBA’s values are not influenced by other aspects of data complexity, a correlation analysis between data complexity measures and imbalance measures’ scores is performed. The correlation with classification performance is also added. The 20 data complexity measures, which are part of the categories feature based, linearity, neighborhood, network, and dimensionality, described by Lorena et al.,^37^ are included. We refer to their paper for more details on these measures. All data complexity measures are computed with the Python pymfe package. Unfortunately, for large datasets (more than 30,000 samples or more than 20,000 samples combined with more than 100 features), the resources needed were too high. Therefore, a sample of 10% of the dataset was taken for the analysis of 6 of the 70 real datasets: adult, connect-4, loc_build, loc_floor, shuttle, and skin.

#### Correlation coefficients, evaluation metrics, and classifiers

The accuracy of an imbalance measure is assessed with the correlation between that imbalance measure’s result and the classification performance.^35^^,^^39^^,^^40^^,^^41^ In all experiments on the synthetic and real datasets, for all imbalance measures, the correlations between results and classification performance are compared. The correlation coefficients, evaluation metrics, and classifiers used to that effect are presented in this subsection.

#### Correlation coefficients

The relationship between imbalance measures and classification performance in both binary and multiclass problems is investigated. While it can be hypothesized that classification becomes increasingly challenging as dataset imbalance grows, it remains unclear whether this relationship is strictly linear or monotonic without necessarily being linear. To investigate both linear and non-linear—albeit monotonic—correlations, we measure both PCC and SRCC.^46^ PCC calculates the strength of linear relationships, whereas SRCC measures non-linear monotonic relationships by using the ranks of values instead of the actual values. Therefore, SRCC is more robust to outliers. If SRCC > PCC, the relation is either monotonic but not linear, or potential outliers are affecting the PCC score. If SRCC < PCC, either the relation is strongly linear or many tied values in the ranks are present, impacting the SRCC score. Lastly, if the relationship is linear and the dataset does not contain significant outliers, PCC and SRCC should yield similar values.

Note that although the different imbalance measures vary in their range (Table 1), PCC and SRCC are scale invariant, ensuring a fair comparison. Similarly, both C1 and IF are inversely scaled compared to other imbalance measures: lower C1 and IF values indicate more imbalanced datasets, whereas higher values indicate more imbalanced datasets for the other measures. As a result, the signs of PCC and SRCC for C1 and IF are opposite compared to the other measures. To compare the strength of the correlation fairly, the absolute values of the correlation coefficients are considered and not their signs.

#### Evaluation metrics

When it comes to imbalanced datasets, there is no consensus on which metric should be preferred, with various papers using or advocating for different ones.^39^^,^^41^^,^^53^ Most of the previous papers introducing an imbalanced measure used the f1-score,^35^^,^^40^^,^^41^ which is one of the most common evaluation metrics in ML for imbalanced classification.^53^ In imbalanced binary problems, the positive and negative classes become synonyms for the minority and majority classes. The f1-score is defined as the harmonic mean between precision (i.e., accuracy of positive predictions) and recall (i.e., detection of real positives). For multiclass problems, the macro average f1-score across classes gives equal importance to all classes, without taking into account their sizes.^53^ However, the f1-score does not include the number of true negatives and focuses only on the prediction of positive labels. In contrast, the geometric mean (g-mean) includes the true negatives by using both sensitivity (i.e., recall or detection of real positives) and specificity (i.e., accuracy of negative predictions). By doing so, it gives importance to recognizing properly both the positive (minority) and the negative (majority) classes, which can result in notable changes from evaluation metrics, like the f1-score, that do not consider true negatives.^53^ Therefore, both the f1-score and g-mean are reported. Similar to the f1-score, the macro average of the g-mean is used for multiclass problems.

#### Classifiers

For all synthetic datasets, the support vector machine (SVM) model from scikit-learn is used with its default parameters, as the SVM represents one of the most selected classifiers in the literature^33^ and appears to be less affected than others by the class-imbalance problem.^32^ Considering that the generation of synthetic datasets—made with the make_classification function from scikit-learn—contains randomness, each scenario is run 100×. PCC and SRCC are calculated at each run; therefore, the mean and standard deviation are reported for each imbalance measure.

For the real datasets, the correlation analysis is performed with 5 different classifiers (taken from scikit-learn), which are common in the literature for supervised ML: SVM, linear discriminant analysis (LDA), random forest (RF), *k*-nearest neighbors (*k*-NNs), and multi-layer perceptron (MLP).^33^ No tuning of the parameters is done, so all datasets share the same ones: for SVM, LDA, RF, and MLP, the scikit-learn default parameters are used. A rule of thumb for the number of neighbors in *k*-NNs is the square root of the number of samples,^54^ which we implement due to the various sizes of the 70 datasets. The use of multiple classifiers, with no tuning of hyperparameters, ensures generalizable results, as opposed to classifier-dependent results, making our extensive benchmark more robust. Compared to the synthetic datasets, no randomness due to synthetic generation is involved; thus, we do not report the mean and standard deviation.

For the correlation between imbalance measures and evaluation metrics, the f1-score or g-mean corresponds to the average f1-score or g-mean obtained from the 5-fold cross-validation.

## Results

The comparison of the 8 imbalance measures, IR, Adj-IR, C1, C2, ID, LRID, IF, and SIMBA, on both synthetic and real data is presented in this section. The results of the ablation study and the data complexity analysis are also included.

### SIMBA follows all expected trends on synthetic data

**Figure 3:**
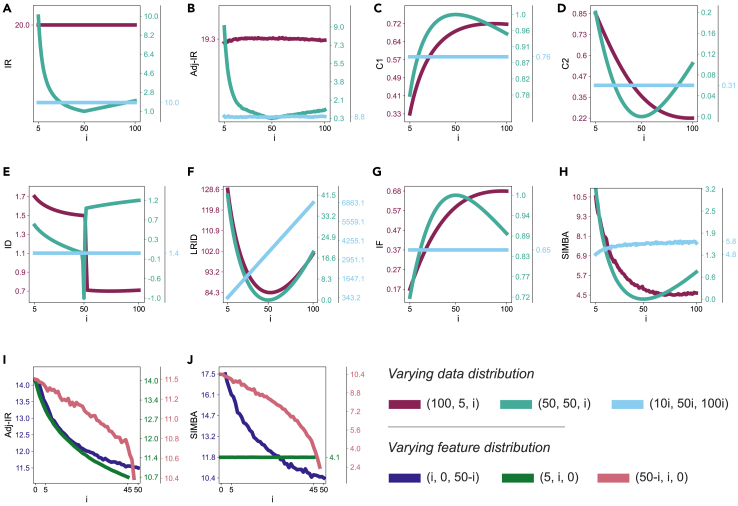
Trends of 8 imbalance measures for datasets with varying data and feature distribution (A–H) Datasets with varying data distribution for (A) imbalance ratio (IR), (B) adjusted imbalance ratio (Adj-IR), (C) entropy of class proportions (C1), (D) multiclass imbalance ratio (C2), (E) imbalance degree (ID), (F) likelihood ratio imbalance degree (LRID), (G) imbalance factor (IF), and (H) status of imbalance (SIMBA). (I and J) Datasets with varying feature distribution for (I) Adj-IR and (J) SIMBA. For data, we consider 3 distributions of 3 classes: (100, 5, *i*), (50, 50, *i*), and (10*i*, 50*i*, 100*i*), with *i* ranging from 5 to 100. For feature components, we consider 3 distributions (informative, redundant, and not informative features): (*i*, 0, 50-*i*) with *i* ranging from 2 to 50, (5, *i*, 0) with *i* ranging from 0 to 45, and (50-*i*, *i*, 0) with *i* ranging from 0 to 48. Note: for readability, the tick labels on the multiple *y* axes are rounded to 1 or 2 decimals.

The trends of all imbalance measures in the 3 scenarios with variations in data distribution and 3 with variations in feature components are shown in Figure 3. The mean and standard deviation of PCC and SRCC between imbalance scores with each measure and classification performance over the 100 runs are reported in Table 4.

**Table 4:** Resulting correlation coefficients on synthetic datasets

	Correlation	IR	Adj-IR	C1	C2	ID	LRID	IF	SIMBA
**Varying data distribution**									

(100, 5, *i*) with range *i*: (5, 100)	PCC with f1-score	–	0.05 (0.29)	0.66 (0.14)	−0.62 (0.13)	−0.46 (0.10)	−0.58 (0.15)	0.64 (0.13)	**−0.75 (0.11)**
	PCC with g-mean	–	0.05 (0.33)	0.74 (0.09)	−0.72 (0.08)	−0.55 (0.08)	−0.62 (0.11)	0.73 (0.09)	**−0.81 (0.07)**
	SRCC with f1-score	–	−0.02 (0.24)	0.49 (0.12)	−0.49 (0.12)	−0.47 (0.13)	−0.29 (0.15)	0.49 (0.12)	**−0.63 (0.11)**
	SRCC with g-mean	–	0.02 (0.28)	0.59 (0.10)	−0.59 (0.10)	−0.57 (0.11)	−0.34 (0.13)	0.59 (0.09)	**−0.71 (0.08)**
(50, 50, *i*) with range *i*: (5, 100)	PCC with f1-score	−0.64 (0.08)	−0.61 (0.08)	0.64 (0.10)	−0.56 (0.12)	0.18 (0.12)	−0.57 (0.11)	0.61 (0.11)	**−0.65 (0.10)**
	PCC with g-mean	−0.54 (0.10)	−0.52 (0.10)	0.55 (0.11)	−0.49 (0.13)	0.16 (0.14)	−0.50 (0.12)	0.53 (0.12)	**−0.57 (0.11)**
	SRCC with f1-score	**−0.36 (0.16)**	−0.34 (0.13)	0.35 (0.16)	−0.33 (0.15)	0.14 (0.16)	−0.32 (0.15)	0.34 (0.16)	−0.35 (0.15)
	SRCC with g-mean	**−0.36 (0.16)**	−0.34 (0.13)	0.35 (0.16)	−0.33 (0.16)	0.12 (0.16)	−0.33 (0.16)	0.35 (0.16)	**−0.36 (0.16)**
(10*i*, 50*i*, 100*i*) with range *i*: (5, 100)	PCC with f1-score	–	0.0 (0.11)	–	–	–	0.16 (0.09)	–	−0.39 (0.11)
	PCC with g-mean	–	0.0 (0.11)	–	–	–	0.17 (0.09)	–	−0.41 (0.10)
	SRCC with f1-score	–	0.0 (0.11)	–	–	–	0.15 (0.09)	–	−0.41 (0.09)
	SRCC with g-mean	–	0.0 (0.11)	–	–	–	0.16 (0.09)	–	

**Varying feature distribution**									

(*i*, 0, 50-*i*) with range *i*: (2, 50)	PCC with f1-score	–	−0.58 (0.15)	–	–	–	–	–	**−0.71 (0.12)**
	PCC with g-mean	–	−0.58 (0.16)	–	–	–	–	–	**−0.72 (0.12)**
	SRCC with f1-score	–	−0.59 (0.13)	–	–	–	–	–	**−0.67 (0.13)**
	SRCC with g-mean	–	−0.59 (0.15)	–	–	–	–	–	**−0.68 (0.14)**
(5, *i*, 0) with range *i*: (0, 45)	PCC with f1-score	–	0.02 (0.25)	–	–	–	–	–	0.05 (0.16)
	PCC with g-mean	–	0.02 (0.27)	–	–	–	–	–	0.04 (0.15)
	SRCC with f1-score	–	0.01 (0.26)	–	–	–	–	–	0.04 (0.17)
	SRCC with g-mean	–	0.01 (0.27)	–	–	–	–	–	0.03 (0.16)
(50-*i*, *i*, 0) with range *i*: (0, 48)	PCC with f1-score	–	−0.69 (0.11)	–	–	–	–	–	**−0.78 (0.08)**
	PCC with g-mean	–	−0.73 (0.10)	–	–	–	–	–	**−0.82 (0.07)**
	SRCC with f1-score	–	−0.67 (0.13)	–	–	–	–	–	**−0.79 (0.07)**
	SRCC with g-mean	–	−0.70 (0.12)	–	–	–	–	–	**−0.82 (0.07)**

Pearson correlation coefficient (PCC) and Spearman’s rank correlation coefficient (SRCC) between measures’ results and evaluation scores—f1-score and g-mean—on synthetic datasets. The eight measures—imbalance ratio (IR), adjusted imbalance ratio (Adj-IR), entropy of class proportions (C1), multiclass imbalance ratio (C2), imbalance degree (ID), likelihood ratio imbalance degree (LRID), imbalance factor (IF), and the status of imbalance (SIMBA)—are compared. Support vector machine (SVM) classifiers are used. When data distribution varies, the number of features is fixed (10 features, among which 2 are informative). When feature components vary, data distribution is fixed to (400, 75, 25). Feature components are divided into informative (info), redundant (rdd), and not informative $\left(\bar{\text{info}}\right)$ features; distributions are written (info, rdd, $\bar{\text{info}}$).

Note: as the generation of synthetic datasets contains randomness, each generation of a dataset was run 100 times. The mean correlation and its standard deviation over the 100 trials are reported with mean (SD). When “–” is written, the imbalance measure was constant on all the runs; therefore, the correlation is null.

For variations in data distribution (Figures 3A–3H), IR and Adj-IR remain constant for all datasets in scenario 1. This is due to IR and Adj-IR only considering the minority class and the majority class, making them inapplicable to multiclass problems. The range of ID changes depending on the number of minority classes (Table 1), which causes a considerable change in the measure’s results in scenarios 1 and 2 when the number of minority classes changes. For this reason, in scenario 2, a dataset with a distribution (50, 50, 51) displays a substantially larger ID than one with a distribution (50, 50, 49), even though they should be similar. LRID’s lack of normalization is clear in scenario 3, where LRID increases as *i* increases, and the dataset gets larger. All these observations are reflected in the PCC and SRCC results in Table 4. C1 and IF display similar results, which is expected since C1 corresponds to one possible version of IF (Table 2). Generally, C1, C2, IF, and SIMBA follow the expected trends in all scenarios, with SIMBA consistently reaching higher values for PCC and SRCC, both with f1-score and g-mean (Table 4). In scenario 3, Figure 3 shows that SIMBA does not remain perfectly constant as *i* increases. This is due to the randomness in the generation of features; as features are taken into account in SIMBA’s formula, the imbalance measure cannot stay constant. Looking at the PCC and SRCC results (Table 4), they are the highest for SIMBA in this scenario, illustrating that including information about features is valuable to measure the extent of imbalance.

For variations in feature components (Figures 3I and 3J), Adj-IR and SIMBA follow similar trends for both scenarios 1 and 3, with SIMBA holding higher PCC and SRCC with both f1-score and g-mean compared to Adj-IR (Table 4). In scenario 2, SIMBA follows the expected trend, remaining constant as redundant features are added. However, Adj-IR decreases as the number of redundant features increases. This is due to Adj-IR integrating the number of discriminant features without looking at redundancy. Nevertheless, both imbalance measures display a near-zero PCC and SRCC with both f1-score and g-mean (Table 4). For SIMBA, this is because it yields almost constant imbalance scores regardless of *i*. For Adj-IR, it is because the variations of resulting imbalance scores are entirely uncorrelated to classification performance.

In short, SIMBA consistently exhibits the expected trends across all scenarios. Specifically, SIMBA reaches higher correlation values compared to other measures when evaluated under varying data distributions and varying feature components. SIMBA appears to incorporate feature information more effectively than Adj-IR, as it results in higher correlation values in the scenarios involving varying feature components. These scenarios’ outcomes suggest that SIMBA provides a more relevant measure of imbalance extent, offering a stronger indication of classification difficulty than any of the other existing imbalance measures. To validate these findings, results with real datasets follow.

### SIMBA outperforms all imbalance measures on real data

**Figure 4:**
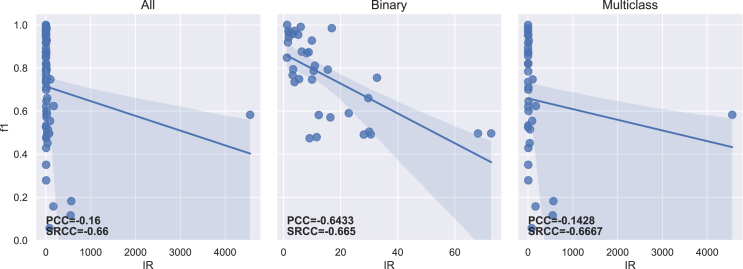
Regression plots between the imbalance ratio and the f1-score The three plots display the regression with all (70), only binary (35), and only multiclass (35) real datasets, respectively. The Pearson correlation coefficient (PCC) and Spearman’s rank correlation coefficient (SRCC) appear on each plot.

**Figure 5:**
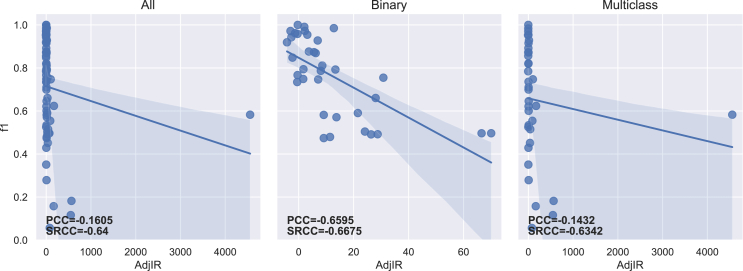
Regression plots between the adjusted imbalance ratio and the f1-score The three plots display the regression with all (70), only binary (35), and only multiclass (35) real datasets, respectively. The Pearson correlation coefficient (PCC) and Spearman’s rank correlation coefficient (SRCC) appear on each plot.

**Figure 6:**
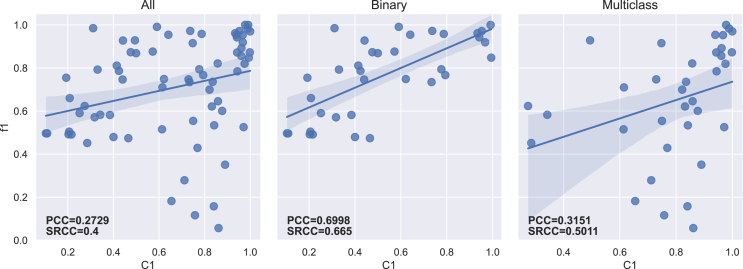
Regression plots between the entropy of class proportions and the f1-score The three plots display the regression with all (70), only binary (35), and only multiclass (35) real datasets, respectively. The Pearson correlation coefficient (PCC) and Spearman’s rank correlation coefficient (SRCC) appear on each plot.

**Figure 7:**
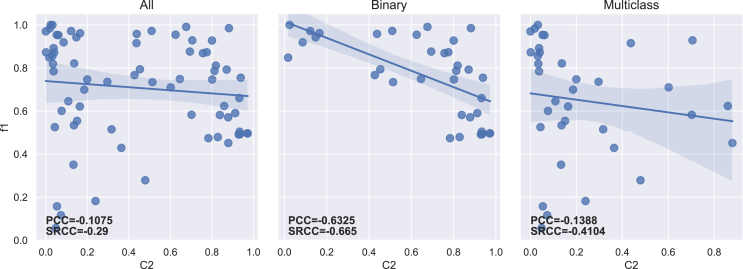
Regression plots between the multiclass imbalance ratio and the f1-score The three plots display the regression with all (70), only binary (35), and only multiclass (35) real datasets, respectively. The Pearson correlation coefficient (PCC) and Spearman’s rank correlation coefficient (SRCC) appear on each plot.

**Figure 8:**
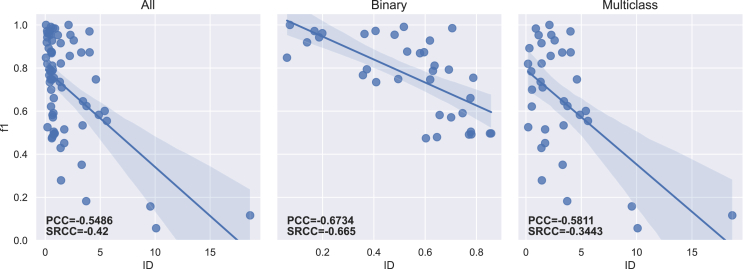
Regression plots between the imbalance degree and the f1-score The three plots display the regression with all (70), only binary (35), and only multiclass (35) real datasets, respectively. The Pearson correlation coefficient (PCC) and Spearman’s rank correlation coefficient (SRCC) appear on each plot.

**Figure 9:**
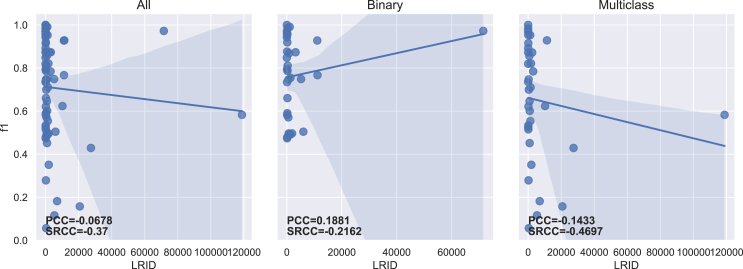
Regression plots between the likelihood ratio imbalance degree and the f1-score The three plots display the regression with all (70), only binary (35), and only multiclass (35) real datasets, respectively. The Pearson correlation coefficient (PCC) and Spearman’s rank correlation coefficient (SRCC) appear on each plot.

**Figure 10:**
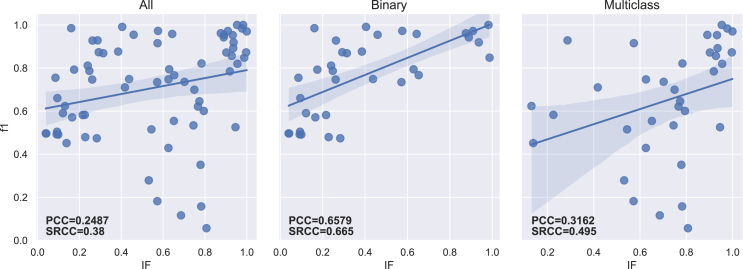
Regression plots between the imbalance factor and the f1-score The three plots display the regression with all (70), only binary (35), and only multiclass (35) real datasets, respectively. The Pearson correlation coefficient (PCC) and Spearman’s rank correlation coefficient (SRCC) appear on each plot.

**Figure 11:**
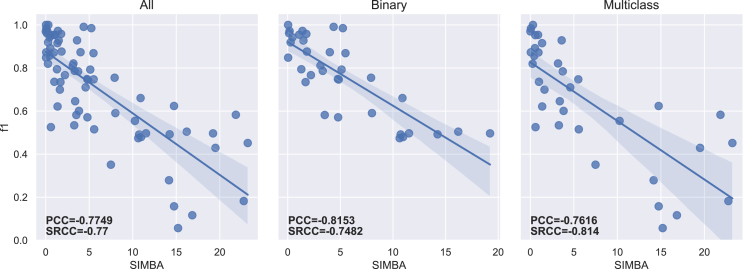
Regression plots between the status of imbalance and the f1-score The three plots display the regression with all (70), only binary (35), and only multiclass (35) real datasets, respectively. The Pearson correlation coefficient (PCC) and Spearman’s rank correlation coefficient (SRCC) appear on each plot.

The PCC and SRCC results between each imbalance measure and both f1-score and g-mean are presented in Table 5 for the 70 datasets. Table 6 displays the results obtained for binary (35) and multiclass (35) datasets separately. The regression lines between f1-scores and each measure’s results are presented in Figures 4, 5, 6, 7, 8, 9, 10, and 11.

**Table 5:** Resulting correlation coefficients on real datasets

Classifier	Correlation	IR	Adj-IR	C1	C2	ID	LRID	IF	SIMBA
SVM	PCC with f1-score	−0.16	−0.16	0.27^a^	−0.11	−0.55^b^	−0.07	0.25^a^	**−0.77**^b^
	PCC with g-mean	−0.10	−0.10	0.46^b^	−0.32^a^	−0.40^b^	−0.01	0.43^b^	**−0.78**^b^
	SRCC with f1-score	−0.66^b^	−0.64^b^	0.40^b^	−0.29^a^	−0.42^b^	−0.37^a^	0.38^a^	**−0.77**^b^
	SRCC with g-mean	−0.68^b^	−0.67^b^	0.53^b^	−0.44^b^	−0.31^a^	−0.37^a^	0.52^b^	**−0.80**^b^
LDA	PCC with f1-score	−0.24^a^	−0.24^a^	0.11	0.03	−0.58^b^	−0.16	0.10	**−0.71**^b^
	PCC with g-mean	−0.16	−0.16	0.27^a^	−0.17	−0.44^b^	−0.10	0.27^a^	**−0.73**^b^
	SRCC with f1-score	−0.55^b^	−0.53^b^	0.25^a^	−0.15	−0.41^b^	−0.33^a^	0.24^a^	**−0.67**^b^
	SRCC with g-mean	−0.56^b^	−0.55^b^	0.38^a^	−0.31^a^	−0.28^a^	−0.35^a^	0.37^a^	**−0.72**^b^
RF	PCC with f1-score	−0.09	−0.09	0.20	−0.05	−0.56^b^	−0.02	0.18	**−0.67**^b^
	PCC with g-mean	−0.04	−0.04	0.38^a^	−0.25^a^	−0.40^b^	0.03	0.35^a^	**−0.67**^b^
	SRCC with f1-score	−0.62^b^	−0.61^b^	0.36^a^	−0.26^a^	−0.37^a^	−0.34^a^	0.35^a^	**−0.69**^b^
	SRCC with g-mean	−0.63^b^	−0.62^b^	0.46^b^	−0.38^a^	−0.25^a^	−0.34^a^	0.45^b^	**−0.70**^b^
*k*-NN	PCC with f1-score	−0.20	−0.20	0.24^a^	−0.07	−0.53^b^	−0.09	0.21	**−0.74**^b^
	PCC with g-mean	−0.11	−0.11	0.43^b^	−0.30^a^	−0.38^a^	−0.01	0.41^b^	**−0.74**^b^
	SRCC with f1-score	−0.62^b^	−0.61^b^	0.31^a^	−0.20	−0.43^b^	−0.31^a^	0.30^a^	**−0.73**^b^
	SRCC with g-mean	−0.64^b^	−0.63^b^	0.47^b^	−0.39^b^	−0.29^a^	−0.3^a^	0.46^b^	**−0.77**^b^
MLP	PCC with f1-score	−0.08	−0.08	0.17	−0.03	−0.58^b^	−0.00	0.16	**−0.71**^b^
	PCC with g-mean	−0.04	−0.04	0.35^a^	−0.23	−0.43^b^	−0.04	0.33^a^	**−0.72**^b^
	SRCC with f1-score	−0.62^b^	−0.60^b^	0.35^a^	−0.26^a^	−0.40^b^	−0.32^a^	0.34^a^	**−0.73**^b^
	SRCC with g-mean	−0.61^b^	−0.60^b^	0.45^b^	−0.38^a^	−0.27^a^	−0.31^a^	0.44^b^	**−0.74**^b^
Mean	PCC with f1-score	−0.15	−0.15	0.20	−0.05	−0.56	−0.07	0.18	**−0.72**
	PCC with g-mean	−0.09	−0.09	0.38	−0.25	−0.41	−0.04	0.36	**−0.73**
	SRCC with f1-score	−0.61	−0.60	0.33	−0.23	−0.41	−0.33	0.32	**−0.72**
	SRCC with g-mean	−0.62	−0.61	0.46	−0.38	−0.28	−0.34	0.45	**−0.75**

Pearson correlation coefficient (PCC) and Spearman’s rank correlation coefficient (SRCC) between the measure’s result and evaluation scores—f1-score and g-mean—on real datasets. The eight measures—imbalance ratio (IR), adjusted imbalance ratio (Adj-IR), entropy of class proportions (C1), multiclass imbalance ratio (C2), imbalance degree (ID), likelihood ratio imbalance degree (LRID), imbalance factor (IF), and the status of imbalance (SIMBA)—are compared. The results on all datasets are presented for 5 different classifiers: SVM, linear discriminant analysis (LDA), random forests (RFs), *k*-nearest neighbors (*k*-NNs), and multi-layer perceptron (MLP).

a For PCC and SRCC, *p* < 0.05.

b For PCC and SRCC, *p* < 0.001.

**Table 6:** Resulting correlation coefficients on real datasets decomposed into binary and multiclass datasets

Classifier	Datasets	Correlation	IR	Adj-IR	C1	C2	ID	LRID	IF	SIMBA
SVM	binary	PCC with f1-score	−0.64^a^	−0.66^a^	0.70^a^	−0.63^a^	−0.67^a^	0.19	0.66^a^	**−0.82**^a^
		PCC with g-mean	−0.65^a^	−0.66^a^	0.72^a^	−0.66^a^	−0.70^a^	0.19	0.68^a^	**−0.81**^a^
		SRCC with f1-score	−0.66^a^	−0.67^a^	0.66^a^	−0.67^a^	−0.66^a^	−0.22	0.66^a^	**−0.75**^a^
		SRCC with g-mean	−0.72^a^	−0.72^a^	0.72^a^	−0.72^a^	−0.72^a^	−0.27	0.72^a^	**−0.78**^a^
	multiclass	PCC with f1-score	−0.14	−0.14	0.32	−0.14	−0.58^a^	−0.14	0.32	**−0.76**^a^
		PCC with g-mean	−0.12	−0.12	0.32	−0.16	−0.57^a^	−0.12	0.32	**−0.78**^a^
		SRCC with f1-score	−0.67^a^	−0.63^a^	0.50^a^	−0.41^b^	−0.34^b^	−0.47^b^	0.49^a^	**−0.81**^a^
		SRCC with g-mean	−0.67^a^	−0.65^a^	0.53^a^	−0.44^b^	−0.35^b^	−0.47^b^	0.52^a^	**−0.83**^a^
LDA	binary	PCC with f1-score	−0.39^b^	−0.42^b^	0.57^a^	−0.56^a^	−0.57^a^	0.15	0.57^a^	**−0.68**^a^
		PCC with g-mean	−0.37^b^	−0.39^b^	0.54^a^	−0.53^a^	−0.54^a^	0.16	0.54^a^	**−0.68**^a^
		SRCC with f1-score	−0.59^a^	−0.60^a^	0.59^a^	−0.59^a^	−0.59^a^	−0.21	0.59^a^	**−0.71**^a^
		SRCC with g-mean	−0.54^a^	−0.54^a^	0.54^a^	−0.54^a^	−0.54^a^	−0.21	0.54^a^	**−0.68**^a^
	multiclass	PCC with f1-score	−0.22	−0.22	0.25	−0.10	−0.55^a^	−0.24	0.26	**−0.75**^a^
		PCC with g-mean	−0.18	−0.18	0.26	−0.13	−0.52^a^	−0.20	0.27	**−0.75**^a^
		SRCC with f1-score	−0.57^a^	−0.55^a^	0.38^b^	−0.31	−0.32	−0.42^b^	0.38^b^	**−0.73**^a^
		SRCC with g-mean	−0.56^a^	−0.55^a^	0.41^b^	−0.35^b^	−0.27	−0.42^b^	0.40^b^	**−0.73**^a^
RF	binary	PCC with f1-score	−0.67^a^	−0.69^a^	0.69^a^	−0.62^a^	−0.66^a^	0.18	0.64^a^	**−0.81**^a^
		PCC with g-mean	−0.67^a^	−0.69^a^	0.70^a^	−0.63^a^	−0.68^a^	0.17	0.66^a^	**−0.81**^a^
		SRCC with f1-score	−0.69^a^	−0.69^a^	0.69^a^	−0.69^a^	−0.69^a^	−0.21	0.69^a^	**−0.75**^a^
		SRCC with g-mean	−0.70^a^	−0.70^a^	0.70^a^	−0.70^a^	−0.70^a^	−0.26	0.70^a^	**−0.77**^a^
	multiclass	PCC with f1-score	−0.06	−0.06	0.11	0.05	**−0.61**^a^	−0.07	0.13	**−0.61**^a^
		PCC with g-mean	−0.05	−0.05	0.10	0.05	−0.59^a^	−0.05	0.12	**−0.61**^a^
		SRCC with f1-score	−0.61^a^	−0.59^a^	0.38^b^	−0.26	−0.38^b^	−0.43^b^	0.37^b^	**−0.66**^a^
		SRCC with g-mean	−0.60^a^	−0.58^a^	0.39^b^	−0.28	−0.35^b^	−0.41^b^	0.39^b^	**−0.66**^a^
*k*-NN	binary	PCC with f1-score	−0.61^a^	−0.63^a^	0.70^a^	−0.63^a^	−0.68^a^	0.24	0.66^a^	**−0.77**^a^
		PCC with g-mean	−0.61^a^	−0.63^a^	0.72^a^	−0.65^a^	−0.69^a^	0.25	0.68^a^	**−0.76**^a^
		SRCC with f1-score	−0.64^a^	−0.65^a^	0.64^a^	−0.64^a^	−0.64^a^	−0.16	0.64^a^	**−0.73**^a^
		SRCC with g-mean	−0.71^a^	−0.71^a^	0.71^a^	−0.71^a^	−0.71^a^	−0.22	0.71^a^	**−0.78**^a^
	multiclass	PCC with f1-score	−0.18	−0.18	0.31	−0.14	−0.52^a^	−0.19	0.31	**−0.74**^a^
		PCC with g-mean	−0.13	−0.13	0.30	−0.16	−0.51^a^	−0.14	0.30	**−0.73**^a^
		SRCC with f1-score	−0.62^a^	−0.61^a^	0.44^b^	−0.34^b^	−0.35^b^	−0.43^b^	0.43^b^	**−0.78**^a^
		SRCC with g-mean	−0.57^a^	−0.56^a^	0.41^b^	−0.33^b^	−0.26	−0.40^b^	0.41^b^	**−0.74**^a^
MLP	binary	PCC with f1-score	−0.53^a^	−0.56^a^	0.63^a^	−0.58^a^	−0.61^a^	0.21	0.60^a^	**−0.79**^a^
		PCC with g-mean	−0.57^a^	−0.59^a^	0.64^a^	−0.59^a^	−0.62^a^	0.20	0.61^a^	**−0.81**^a^
		SRCC with f1-score	−0.64^a^	−0.65^a^	0.64^a^	−0.64^a^	−0.64^a^	−0.15	0.64^a^	**−0.74**^a^
		SRCC with g-mean	−0.64^a^	−0.65^a^	0.64^a^	−0.64^a^	−0.64^a^	−0.21	0.64^a^	**−0.76**^a^
	multiclass	PCC with f1-score	−0.05	−0.05	0.16	−0.01	−0.62^a^	−0.05	0.18	**−0.68**^a^
		PCC with g-mean	−0.04	−0.04	0.17	−0.04	−0.59^a^	−0.03	0.19	**−0.69**^a^
		SRCC with f1-score	−0.63^a^	−0.60^a^	0.41^b^	−0.33^b^	−0.37^b^	−0.43^b^	0.42^b^	**−0.75**^a^
		SRCC with g-mean	−0.61^a^	−0.59^a^	0.44^b^	−0.36^b^	−0.31	−0.40^b^	0.45^b^	**−0.75**^a^

Pearson correlation coefficient (PCC) and Spearman’s rank correlation coefficient (SRCC) between measures’ results and evaluation scores—f1-score and g-mean—on binary real datasets and multiclass real datasets, separately. The eight measures—imbalance ratio (IR), adjusted imbalance ratio (Adj-IR), entropy of class proportions (C1), multiclass imbalance ratio (C2), imbalance degree (ID), likelihood ratio imbalance degree (LRID), imbalance factor (IF), and status of imbalance (SIMBA)—are compared. The results on all datasets are presented for 5 different classifiers: support vector machine (SVM), linear discriminant analysis (LDA), random forests (RFs), *k*-nearest neighbors (*k*-NNs), and multi-layer perceptron (MLP).

a For PCC and SRCC, *p* < 0.001.

b For PCC and SRCC, *p* < 0.05.

IR, Adj-IR, and LRID do not have a significant linear relationship with classification performance (Table 5). C1, C2, and IF show a significant linear relationship with classification performance but not across all classifiers or evaluation metrics. ID and SIMBA are the only two metrics consistently displaying a significant linear relationship with classification performance across all classifiers and both evaluation metrics.

SIMBA consistently reaches higher correlations, regardless of the classifier used (Table 5). It shows robustness to the number of classes in datasets by performing better than other imbalance measures for both binary and multiclass datasets (Table 6). For PCC, ID comes second, while IR and Adj-IR are in second position for SRCC. However, IR and Adj-IR have very low PCCs. The fact that IR and Adj-IR have a much larger SRCC than PCC indicates that there exists a monotonic relationship between classification scores and both IR and Adj-IR scores, albeit not a linear one. This confirms the findings of Thabtah et al.^12^

As expected, Table 6 and Figures 4 and 5 show that IR and Adj-IR perform well on binary datasets (PCCs between 0.64 and 0.66) but fail on the multiclass ones (PCC of 0.14). Adj-IR performs better than IR on binary datasets, which supports the findings of Zhu et al.^35^ ID works fairly well on multiclass datasets compared to IR, Adj-IR, C1, C2, LRID, and IF. However, contrary to SIMBA, the regression line is very different between binary and multiclass datasets (Figure 8), leading to a poorer result when both are considered together.

The lack of normalization for LRID is evident in Figure 9, with one dataset reaching a value of approximately 120,000. Once again, C1 and IF have very close results, as C1 corresponds to one possible version of IF (Table 2; Figures 6 and 10). Both measures have strong PCCs for binary datasets but substantially lower PCCs for multiclass ones, especially in comparison to ID and SIMBA (Table 6). On average, C1 (Shannon version of IF) performs slightly better than IF (Collision version). This contradicts the findings of Pirizadeh et al.,^41^ who found the Collision version to perform better on average. This is surely due to the significantly smaller number of datasets used in that study. C2 also fails to capture classification difficulty on multiclass datasets (Figure 7). Finally, SIMBA displays similar regression lines for both binary and multiclass datasets (Figure 11) and achieves the best correlation results in all cases. With a mean PCC and SRCC ranging between 0.72 and 0.75, with f1-score and g-mean, respectively, SIMBA’s values present a strong correlation with the classification performance.^46^ These results confirm the findings obtained with the synthetic datasets.

### Two of SIMBA’s core components are key to quantify imbalance

**Figure 12:**
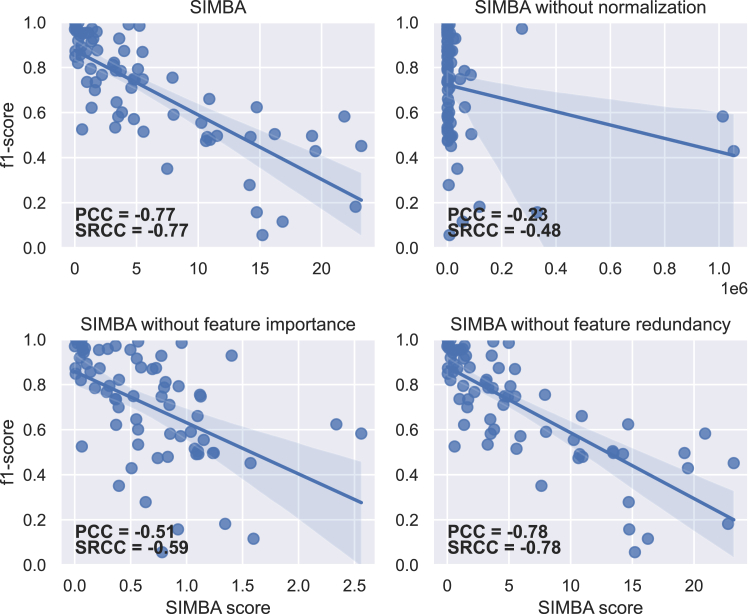
Regression plots between 4 versions of SIMBA and the f1-score on all real datasets The 4 versions consist of SIMBA entirely, SIMBA without normalization, SIMBA without feature importance, and SIMBA without considering feature redundancy. The Pearson correlation coefficient (PCC) and Spearman’s rank correlation coefficient (SRCC) appear on each plot.

The results of the ablation study display the impact of each core component—namely, normalization, feature importance, and feature redundancy—of SIMBA’s formula on the PCC and SRCC results (Figure 12). Disregarding feature redundancy does not impact the results for PCC and SRCC, with even a slight increase of 0.01. In contrast, without normalization or feature importance, the results for PCC and SRCC substantially decrease.

Without normalization, SIMBA resembles LRID, for which large datasets lead to extremely high values, making them seem more imbalanced than others but only because of their size (Figure 9). This results in a drop of 0.54 and 0.26 for PCC and SRCC, respectively.

Without feature importance, SIMBA loses information on the classification difficulty in the case of imbalance. Some datasets, even though they present an imbalance, can remain easy to classify if the features are sufficiently discriminant. This is shown in Figure 12, where datasets that are easy to classify (f1-score ≥ 0.8) have SIMBA values spreading over half the SIMBA range on the 70 datasets when disregarding feature importance. In comparison, when SIMBA is taken as a whole, SIMBA values for these datasets cover only the lower fifth of the SIMBA range, indicating their ease of classification. This results in a drop of 0.26 and 0.18 for PCC and SRCC, respectively.

### SIMBA is independent of other data complexity measures

The results of the data complexity analysis (Table 7) show that no strong correlation exists between SIMBA and data complexity measures. The only significant correlations (at best, moderate correlations^46^) appear within the feature-based category, which is coherent, as SIMBA includes information about features.

**Table 7:** Results of the data complexity analysis

Category	Measure	#DS	IR	Adj-IR	C1	C2	ID	LRID	IF	SIMBA	f1-score
Feature-based	maximum Fisher’s discriminant ratio (F1)	63	−0.05	−0.04	−0.46^a^	0.50^a^	−0.30^b^	0.01	−0.46^a^	0.34^b^	−0.35^b^
	directional-vector maximum Fisher’s discriminant ratio (F1v)	69	−0.02	−0.02	−0.05	0.07	−0.07	0.06	−0.07	0.40^a^	−0.56^a^
	volume of overlapping region (F2)	57	−0.08	−0.08	0.20	−0.22	−0.09	0.01	0.22	−0.15	−0.06
	maximum individual feature efficiency (F3)	70	0.07	0.07	0.20	−0.33^b^	0.70^a^	0.06	0.21	0.43^a^	−0.65^a^
	collective feature efficiency (F4)	70	−0.10	−0.10	0.05	0.01	−0.15	0.04	0.02	−0.00	−0.20
Linearity	sum of the error distance by linear programming (L1)	70	−0.09	−0.09	0.20	−0.15	−0.04	0.08	0.16	0.13	−0.38^b^
	error rate of linear classifier (L2)	70	−0.08	−0.08	0.24^b^	−0.19	−0.04	0.03	0.20	0.08	−0.36^b^
	non-linearity of a linear classifier (L3)	70	−0.07	−0.07	0.25^b^	−0.19	−0.02	0.04	0.21	0.09	−0.38^b^
Neighborhood	fraction of borderline points (N1)	70	−0.03	−0.03	0.40^a^	−0.50^a^	0.61^a^	−0.08	0.40^a^	0.25^b^	−0.66^a^
	ratio of intra-/extra-class nearest-neighbor distance (N2)	62	−0.24	−0.24	0.31^b^	−0.40^b^	0.49^a^	−0.34^b^	0.32^b^	0.22	−0.54^a^
	error rate of the nearest-neighbor classifier (N3)	70	−0.01	−0.01	0.33^b^	−0.43^a^	0.69^a^	−0.05	0.32^b^	0.31^b^	−0.71^a^
	non-linearity of the nearest-neighbor classifier (N4)	70	0.07	0.07	0.56^a^	−0.64^a^	0.58^a^	0.01	0.56^a^	0.10	−0.41^a^
	fraction of hyperspheres covering data (T1)	70	−0.06	−0.06	0.47^a^	−0.57^a^	0.62^a^	−0.12	0.47^a^	0.24^b^	−0.61^a^
	local set average cardinality (LSC)	70	0.02	0.02	0.45^a^	−0.50^a^	0.26^b^	0.02	0.46^a^	0.10	−0.30^b^
Network	average density of the network (density)	70	0.04	0.04	0.62^a^	−0.72^a^	0.61^a^	0.01	0.62^a^	0.02	−0.37^b^
	clustering coefficient (ClsCoef)	70	−0.07	−0.07	−0.23	0.25^b^	−0.22	−0.11	−0.24^b^	0.13	−0.21
	hub score (Hubs)	70	−0.08	−0.08	0.71^a^	−0.79^a^	0.49^a^	−0.11	0.72^a^	−0.11	−0.17
Dimensionality	average number of features per dimension (T2)	70	−0.07	−0.07	0.21	−0.20	−0.06	−0.11	0.23	−0.19	0.21
	average number of PCA dimensions per point (T3)	70	−0.09	−0.09	0.20	−0.18	−0.09	−0.14	0.20	−0.20	0.14
	ratio of the PCA dimension to the original dimension (T4)	70	−0.12	−0.12	−0.01	0.01	−0.07	−0.07	−0.02	−0.04	−0.07

Pearson correlation coefficients between 20 data complexity measures of 5 categories, as described by Lorena et al.,^37^ and imbalance measures and f1-scores. The correlations with 8 imbalance measures—imbalance ratio (IR), adjusted imbalance ratio (Adj-IR), entropy of class proportions (C1), multiclass imbalance ratio (C2), imbalance degree (ID), likelihood ratio imbalance degree (LRID), imbalance factor (IF), and status of imbalance (SIMBA)—are compared. All measures are calculated with the pymfe library from Python. When data complexity measures returned NaN (not a number) values for a dataset, this dataset was excluded from the correlation analysis. The number of datasets (#DS) included in the correlation analysis is indicated for each data complexity measure.

a *p* < 0.001.

b *p* < 0.05.

C1 and C2 are used as data complexity measures of the class-imbalance category.^37^ Both of them count more significant correlations with other data complexity measures than SIMBA yet weaker correlations with classification performance (Table 5). Hence, SIMBA indicates classification difficulty better while being more independent of other data complexity measures.

Higher values for each of the complexity measures indicate a more complex problem and, in turn, a more difficult classification task. The f1-score is indeed negatively impacted (Table 7). Nonetheless, none of the complexity measures reaches a higher PCC score with classification performance than SIMBA. Thus, SIMBA indicates classification difficulty better than any of the 20 data complexity measures.

## Discussion

Imbalance is omnipresent in real-world data, including in critical domains such as healthcare and finance^7^^,^^8^ (Figure 1). As the performance of modern ML models is highly dependent on the data they were trained with,^55^ imbalance constitutes a considerable challenge. It creates biases toward majority classes, generates unreliable or even incorrect learned patterns for minority classes, and ultimately leads to unfair decision-making.^1^^,^^14^^,^^16^ Notably, it increases the cases of false negatives, which, in certain domains, can lead to dire consequences, for example, determining that a patient with cancer is healthy, leaving them without treatment.^11^

Research has been conducted for decades on how to solve the issue of imbalance and reduce its negative effects on classification.^13^^,^^20^ These solutions are often dependent on the severity of the imbalance. However, so far, there has been no universally shared definition of what constitutes low, moderate, high, or severe imbalance.^30^ The most common imbalance measure is the IR, but it only considers the minority and the majority classes. Consequently, it fails to capture the increased difficulty of imbalance in multiclass problems. Several other measures appeared for imbalance in multiclass problems, but all of them showed limitations (Figure 2). The most recent imbalance measure is the IF.^41^ Our extensive benchmark showed that IF fails to generalize to other datasets than the ones it was introduced with and, thus, does not display a strong correlation with classification difficulty.

None of the existing imbalance measures for multiclass problems took into account data overlap, which has been shown to increase the impact of data imbalance on classification.^14^ To fill this gap, we introduced the imbalance measure SIMBA, which works both for binary and multiclass datasets, considering both data distribution and data overlap. Contrary to other measures, such as Adj-IR, ID, and IF, SIMBA’s formula does not involve any parameter left for the researcher to choose, ensuring more accurate comparisons among researchers. Experiments on synthetic datasets followed by an extensive benchmark on 70 real datasets with 5 ML classifiers showed that SIMBA generalizes well to different classifiers, evaluation metrics, and datasets of varying feature components and sizes. SIMBA consistently outperformed all other imbalance measures. We conclude that SIMBA is robust to the use of various classifiers, works with datasets with any number of classes and features, and gives an accurate indication of classification difficulty, making it a generalizable imbalance measure.

Three core components are included in SIMBA’s formula: (1) normalization, (2) feature importance, and (3) feature redundancy. An ablation study showed that removing the feature redundancy aspect did not degrade the correlation with classification performance, even though the behavior of SIMBA seemed more coherent with this aspect on synthetic datasets. This result emphasizes the importance of validation on real-world, out-of-distribution datasets.^15^ It is possible that the threshold chosen to consider that two features are redundant (a strong correlation of at least 0.9) is not the optimal threshold, and an empirical study in future work could investigate this aspect further. In contrast, removing either of the first two components substantially reduced the correlation between SIMBA and classification performance, highlighting the crucial part these elements play in the calculation of the SIMBA score.

SIMBA quantifies the negative impact of class imbalance on classification performance and considers how this effect increases in the presence of data overlap.^14^^,^^24^ Nevertheless, SIMBA does not cover all the aspects of the data complexity domain. For instance, a perfectly balanced dataset that contains non-discriminant features, and therefore is difficult to classify, will result in a SIMBA value of 0, as the classification difficulty does not stem from imbalance. A data complexity analysis showed that SIMBA is independent of other data complexity measures but indicates classification difficulty better. As such, SIMBA constitutes a measure that describes a specific facet of data complexity, namely the influence of imbalance, and can be used as a data complexity measure for the category class imbalance.^37^ For a complete data complexity analysis, SIMBA should be used with additional data complexity measures.

The accurate quantification of the extent of imbalance in any dataset is now enabled. SIMBA indicates the difficulty of the classification task at hand. Future research can dive into what solutions should be applied to a dataset based on its SIMBA score. Researchers in the field could focus on the creation of a general rule-based algorithm to tackle the imbalance and reduce, or possibly even remove, its negative impacts on classification. With SIMBA, a robust and generalizable measure of data imbalance, one of the core challenges of modern ML, is mitigated.

## Resource availability

### Lead contact

Requests for further information and resources should be directed to and will be fulfilled by the lead contact, Julie R. Pivin-Bachler (julie@pivin-bachler.eu).

### Materials availability

This study did not generate new unique reagents.

### Data and code availability

This paper analyzes existing, publicly available data from the UCI Machine Learning Repository^49^ and the KEEL Dataset Repository.^48^ All the processed datasets and original code needed to evaluate the conclusions in the paper have been deposited at Zenodo^56^ and are publicly available as of the date of publication.

## Acknowledgments

The authors thank the Honda Research Institute in Japan for funding this research.

## Author contributions

J.R.P.-B. conceived, designed, and performed the experiments, analyzed the data, and wrote the paper. E.L.v.d.B. conceived and designed the experiments and wrote the paper.

## Declaration of interests

The authors declare that they have no competing interests.

## Supplemental information

Document S1. Table S1

Document S2. Article plus supplemental information
