# Supplementary material for: SIMBA: A robust and generalizable measure of data imbalance
Source: Patterns (N Y). 2025 Oct 21;6(12):101395. doi: 10.1016/j.patter.2025.101395 (PMC12745995; doi:10.1016/j.patter.2025.101395)
Supplement: Document S1. Table S1 [file mmc1.pdf]

Patterns, Volume *6*

Supplemental information

SIMBA: A robust and generalizable measure of data imbalance

Julie R. Pivin-Bachler and Egon L. van den Broek

Supplemental Information 1 - Detail on the 70 real datasets

This Supplemental Information contains more detail about the 70 real datasets used in this paper. Table S1 gives the frequency of each class in the datasets.

Table S1: Detailed description of the class frequencies of the 70 datasets used in the study.

Dataset	#c	#min	Size	Class frequencies (%)
abalone	28	20	4177	[0.02, 0.02, 0.36, 1.36, 2.75, 6.2, 9.36, 13.6, 16.5, 15.18, 11.66, 6.39, 4.86, 3.02, 2.47, 1.6, 1.39, 1.01, 0.77, 0.62, 0.34, 0.14, 0.22, 0.05, 0.02, 0.02, 0.05, 0.02]
abalone$_{20}$[a]	2	1	1916	[98.64, 1.36]
abalone$_{9-18}$	2	1	731	[94.25, 5.75]
adult	2	1	48842	[76.07, 23.93]
balance	3	1	625	[46.08, 7.84, 46.08]
banknote	2	1	1372	[55.54, 44.46]
bankruptcy	2	1	6819	[96.77, 3.23]
breastcancer	2	1	569	[62.74, 37.26]
cardio$_{10}$	10	6	2126	[18.06, 27.23, 2.49, 3.81, 3.39, 15.62, 11.85, 5.03, 3.25, 9.27]
cardio$_{3}$	3	2	2126	[77.85, 13.88, 8.28]
chess	18	10	28056	[0.1, 0.28, 0.88, 0.29, 0.71, 1.68, 2.11, 2.43, 5.11, 6.1, 7.08, 10.17, 12.82, 14.95, 16.23, 7.72, 1.39, 9.97]
cleveland$_{0_vs_4}$	2	1	173	[92.49, 7.51]
connect-4	3	2	67557	[65.83, 9.55, 24.62]
contraceptive	3	1	1473	[42.7, 22.61, 34.69]
credit	2	1	653	[45.33, 54.67]
dermatology	6	3	358	[31.01, 16.76, 19.83, 13.41, 13.4, 5.59]
dermatology$_{6}$	2	1	358	[94.41, 5.59]
drybean	7	4	13611	[14.89, 9.71, 3.84, 11.98, 14.17, 19.37, 26.05]
ecoli	8	5	336	[42.56, 22.92, 0.6, 0.6, 10.42, 5.95, 1.49, 15.48]
ecoli$_{0}$[b]	2	1	336	[91.37, 8.63]
ecoli$_{1}$	2	1	336	[77.08, 22.92]
glass	6	4	214	[32.71, 35.51, 7.94, 6.07, 4.21, 13.55]
glass$_{0-1-5_vs_2}$	2	1	172	[90.12, 9.88]
glass$_{2}$	2	1	214	[92.06, 7.94]
glass$_{4}$	2	1	214	[93.93, 6.07]
glass$_{5}$	2	1	214	[95.79, 4.21]
glass$_{6}$	2	1	214	[86.45, 13.55]
hayes-roth	3	1	132	[38.64, 38.64, 22.73]

Table S1: Detailed description of the class frequencies of the 70 datasets used in the study.

Dataset	#c	#min	Size	Class frequencies (%)
htru2	2	1	17898	[90.84, 9.16]
ionosphere	2	1	351	[64.1, 35.9]
knowledge	4	1	403	[12.41, 32.01, 30.27, 25.31]
landsat	6	3	6435	[23.82, 10.92, 21.1, 9.73, 10.99, 23.43]
led7digit[c]	2	1	443	[91.65, 8.35]
lenses	3	2	24	[16.67, 20.83, 62.5]
loc_build	3	2	21048	[27.48, 26.15, 46.37]
loc_floor	5	1	21048	[21.38, 25.96, 22.44, 24.8, 5.42]
lymphography	4	2	148	[1.35, 54.73, 41.22, 2.7]
new-thyroid	3	2	215	[69.77, 16.28, 13.95]
new-thyroid$_1$	2	1	215	[83.72, 16.28]
obesity	7	5	2111	[13.6, 13.74, 13.74, 16.63, 12.88, 14.07, 15.35]
page-blocks$_0$	2	1	5472	[89.78, 10.22]
pageblocks	5	4	5473	[89.77, 6.01, 0.51, 1.61, 2.1]
penbased	10	5	1100	[10.45, 10.36, 10.36, 9.64, 10.36, 9.64, 9.55, 10.45, 9.55, 9.64]
poker$_{9_vs_7}$	2	1	244	[96.72, 3.28]
purchase	2	1	12330	[84.53, 15.47]
room	4	3	10129	[81.23, 4.53, 7.38, 6.85]
segment$_0$	2	1	2308	[85.75, 14.25]
shuttle	7	5	58000	[78.6, 0.09, 0.29, 15.35, 5.63, 0.02, 0.02]
skin	2	1	245057	[20.75, 79.25]
soybean	4	3	47	[21.28, 21.28, 21.28, 36.17]
spambase	2	1	4601	[60.6, 39.4]
spect-heart	2	1	267	[20.6, 79.4]
steel	7	4	1941	[8.14, 9.79, 20.14, 3.71, 2.83, 20.71, 34.67]
student	17	11	649	[2.31, 0.15, 0.15, 0.46, 1.54, 5.39, 5.39, 14.95, 16.02, 11.09, 12.63, 9.71, 7.55, 5.55, 4.47, 2.31, 0.31]
theorem	6	4	6118	[41.75, 17.8, 7.94, 12.23, 10.08, 10.2]
thyroid	3	2	720	[2.36, 5.14, 92.5]
vehicle$_0$	2	1	846	[76.48, 23.52]
vowel$_0$	2	1	988	[90.89, 9.11]
wallfollowing	4	2	5456	[40.41, 38.43, 6.01, 15.15]
webphishing	3	1	1353	[51.88, 7.61, 40.5]
wholesale	3	2	440	[17.5, 10.68, 71.82]

Table S1: Detailed description of the class frequencies of the 70 datasets used in the study.

Dataset	#c	#min	Size	Class frequencies (%)
wine	3	2	178	[33.15, 39.89, 26.97]
wine-quality	7	4	6497	[0.46, 3.32, 32.91, 43.65, 16.61, 2.97, 0.08]
winequality$_{red}$[d]	2	1	691	[98.55, 1.45]
wisconsin	2	1	683	[65.01, 34.99]
yeast	10	6	1484	[16.44, 28.91, 31.2, 2.96, 2.36, 3.44, 10.98, 2.02, 1.35, 0.34]
yeast$_{1-2-8-9_vs_7}$	2	1	947	[96.83, 3.17]
yeast$_3$	2	1	1484	[89.02, 10.98]
yeast$_4$	2	1	1484	[96.56, 3.44]
yeast$_5$	2	1	1484	[97.04, 2.96]

All datasets are from either the UCI Machine Learning Repository and KEEL datasets repository, cf. Table 3. The number of classes (#c), minority classes (#min), and the frequency (%) of appearance of each class in the dataset are reported.

[a] Full name: abalone$_{20_vs_8,9,10}$

[b] Full name: ecoli$_{0,1,4,7_vs_2,3,5,6}$

[c] Full name: led7digit$_{0,2,4,5,6,7,8,9_vs_1}$

[d] Full name: winequality$_{red-3_vs_5}$
